# Haplotype associated RNA expression (HARE) improves prediction of complex traits in maize

**DOI:** 10.1371/journal.pgen.1009568

**Published:** 2021-10-04

**Authors:** Anju Giri, Merritt Khaipho-Burch, Edward S. Buckler, Guillaume P. Ramstein

**Affiliations:** 1 Institute of Genomic Diversity, Cornell University, Ithaca, New York; 2 Plant Breeding and Genetics Section, Cornell University, Ithaca, New York; 3 U.S. Department of Agriculture-Agricultural Research Service, Ithaca, New York; "USDA-ARS Pacific West Area", UNITED STATES

## Abstract

Genomic prediction typically relies on associations between single-site polymorphisms and traits of interest. This representation of genomic variability has been successful for predicting many complex traits. However, it usually cannot capture the combination of alleles in haplotypes and it has generated little insight about the biological function of polymorphisms. Here we present a novel and cost-effective method for imputing *cis* haplotype associated RNA expression (HARE), studied their transferability across tissues, and evaluated genomic prediction models within and across populations. HARE focuses on tightly linked *cis* acting causal variants in the immediate vicinity of the gene, while excluding *trans* effects from diffusion and metabolism. Therefore, HARE estimates were more transferrable across different tissues and populations compared to measured transcript expression. We also showed that HARE estimates captured one-third of the variation in gene expression. HARE estimates were used in genomic prediction models evaluated within and across two diverse maize panels–a diverse association panel (Goodman Association panel) and a large half-sib panel (Nested Association Mapping panel)–for predicting 26 complex traits. HARE resulted in up to 15% higher prediction accuracy than control approaches that preserved haplotype structure, suggesting that HARE carried functional information in addition to information about haplotype structure. The largest increase was observed when the model was trained in the Nested Association Mapping panel and tested in the Goodman Association panel. Additionally, HARE yielded higher within-population prediction accuracy as compared to measured expression values. The accuracy achieved by measured expression was variable across tissues, whereas accuracy by HARE was more stable across tissues. Therefore, imputing RNA expression of genes by haplotype is stable, cost-effective, and transferable across populations.

## Author summary

Genomic marker data is widely used in the prediction of many traits. However, prediction has been primarily carried out within populations and without explicit modeling of RNA or protein expression. In this study, we explored the prediction of field traits within and across populations using estimated RNA expression attributable to only the DNA sequence around a gene. We showed that the estimated RNA expression was more transferable across populations and tissues than measured RNA expression. We improved prediction of field traits up to 15% using estimated gene expression as compared to observed expression or gene sequence alone. Overall, these findings indicate that structural and functional information in the gene sequence is highly transferable.

## Introduction

Genomic prediction is a powerful tool to predict quantitative traits using genomic information. In genomic prediction models, genome-wide predictors are incorporated in the model in an attempt to capture variation from all quantitative trait loci (QTL) associated with the quantitative trait [1]. Genome-wide predictors could be single nucleotide polymorphisms (SNPs), haplotypes, or any downstream intermediate responses such as transcriptomes or metabolomes [1–6]. Haplotype sometimes yield higher prediction accuracy when compared to SNPs as they can capture local epistatic effects, can be in tight linkage with the QTL, and can better capture ancestral (identity by descent) relationships [7–10]. Haplotype-based models may be more useful as beneficial haplotypes are conserved across generations due to tight linkage. Downstream responses like gene expression may be biologically “closer” to the phenotype as they reflect transcription processes in different tissues. However, transcription is greatly affected by tissue, time, and growing conditions; therefore, transcriptome information from different tissues has varying power to predict phenotypes [2,4].

Gene expression is a complex phenomenon involving interactions between DNA, cell components, and the environment. Although every tissue in a plant contains the same genomic sequence, gene expression varies widely across tissues producing numerous phenotypes. The variation in gene expression is due to the differences in regulatory regions and regulatory genes. Discerning the role of different factors contributing to expression is a challenge; however, a common approach to analyzing expression is to partition it into *cis* and *trans* components. The *cis* components are polymorphisms linked to the gene, whereas the *trans* components are everything else not directly linked to the gene of interest [11]. *Trans* components can be impacted by polymorphisms arising anywhere in the genome and affect gene expression by the products from diffusion and metabolism [12]. Like many eukaryotes, the expression of any maize gene is often impacted by dozens of transcription factors encoded in *trans* all across the genome [13]. Therefore, *trans* components frequently explain more variation in expression than *cis* components.

Different approaches exist to partition the variation in expression and infer the contribution to expression by *cis* factors only. These include hybrid crosses between inbreds and different testers to partition out background variation from *trans* [11,14], or analyses of genomic sequence linked to genes [15]. Here, we used haplotypes in the gene region and partitioned variation in expression contributed by the *cis* haplotype. Grundberg et al. [16] found that 90% of *cis* variants were shared across plants growing in different environmental conditions and only a few *cis* variants were environment specific as opposed to *trans* variants. The *cis* component of variation is less sensitive to genetic and environmental perturbation, so, they can be stable across different contexts and biological replicates. Partitioning out the variation due to *trans* from overall expression allowed us to get expression effect associated with the *cis* haplotype. We called this transferrable portion of the gene expression as the *cis H*aplotype Associated RNA Expression (HARE). We hypothesized that HARE would be more transferable across tissues than total measured transcript expression. Moreover, the consistent functional and structural information in HARE would result in more accurate prediction than total measured expression in predicting many complex traits.

We used maize to study transferability across different systems (tissues and populations) as it is an important cereal crop and an excellent model system for quantitative genetic studies [17]. Maize’s genotypic and phenotypic diversity has been explored in several studies using different mapping populations, uncovering thousands of genotypes and traits [18]. One example is the Goodman Association panel, which represents the global diversity of inbred genotypes from public maize breeding programs, including approximately 280 genotypes from tropical and temperate regions, sweet corn, and popcorn lines [19]. The Nested Association Mapping panel (NAM) includes a set of approximately 5,000 recombinant inbred lines developed from 25 diverse inbreds crossed to a common parent, B73 [20,21]. NAM captures a large proportion of diversity in maize with less confounding by population structure, compared to diverse samples like in the Goodman Association panel. Both populations have been extensively genotyped and phenotyped for complex traits [22–25]. In addition, the Goodman Association panel also has a large set of available expression data from diverse tissues [26]. Recently these populations were used in the development of a Practical Haplotype Graph (PHG) utilizing high-quality assemblies of NAM founder lines [27]. The PHG summarizes the diversity of these lines as a collection of haplotypes in a graph [27]. In diverse species like maize, with rich allelic series, a wide range of possible alleles might result in the same molecular outcome (e.g., gene expression, protein expression, etc.), a process known as equifinality. HARE can parsimoniously summarize a large series of allelic variants including causal variants, resulting in transferability across populations. Therefore, we hypothesized that HARE would be functionally relevant beyond genomic relationships and would result in higher prediction accuracy than haplotype structure when used to predict many complex traits within and across populations.

To test these hypothesis, we designed a novel method of imputing expression associated with haplotypes in the genic regions by HARE and studied the transferability of imputed expression across tissues and populations. The HARE estimates were imputed in NAM founder’s genic haplotypes using gene expression data previously collected in 7 diverse tissues [26]. The objectives of the study were to: i) partition gene expression variation into *cis* and *trans* components, ii) impute HARE in NAM and the Goodman Association panels based on the shared NAM founder’s haplotypes, iii) assess prediction of many complex traits by using HARE, randomly permuted HARE (preserving haplotype structure only), and measured expression within and across populations, and iv) integrate HARE from different tissues to predict complex phenotypes within and across populations.

## Results

### Phenotypic and genetic diversity in NAM and the Goodman Association panel

The phenotypic distribution of 26 diverse traits is presented in S1 Fig, where the average trait value was higher in 18 traits in NAM than in the Goodman Association panel. The haplotype frequency was also variable across these two panels (S2A and S2B Fig). The median haplotype frequency across genic reference regions was 100 in NAM whereas it was 8 in the Goodman panel. The majority of haplotypes were present in 100 lines as expected from biparental populations with 200 inbred progenies in NAM. Each reference region in NAM was dominated by haplotypes from the common parent (B73), representing half of the haplotypes. We also calculated haplotype entropy from haplotype frequency in each reference region. Haplotype entropy reflects the average information content of haplotype variation in reference regions. As expected, we observed a higher median entropy of 3.03 in the Goodman panel when compared to 2.3 in NAM (S2C and S2D Fig).

### Variance partition in expression

**Fig 1:**
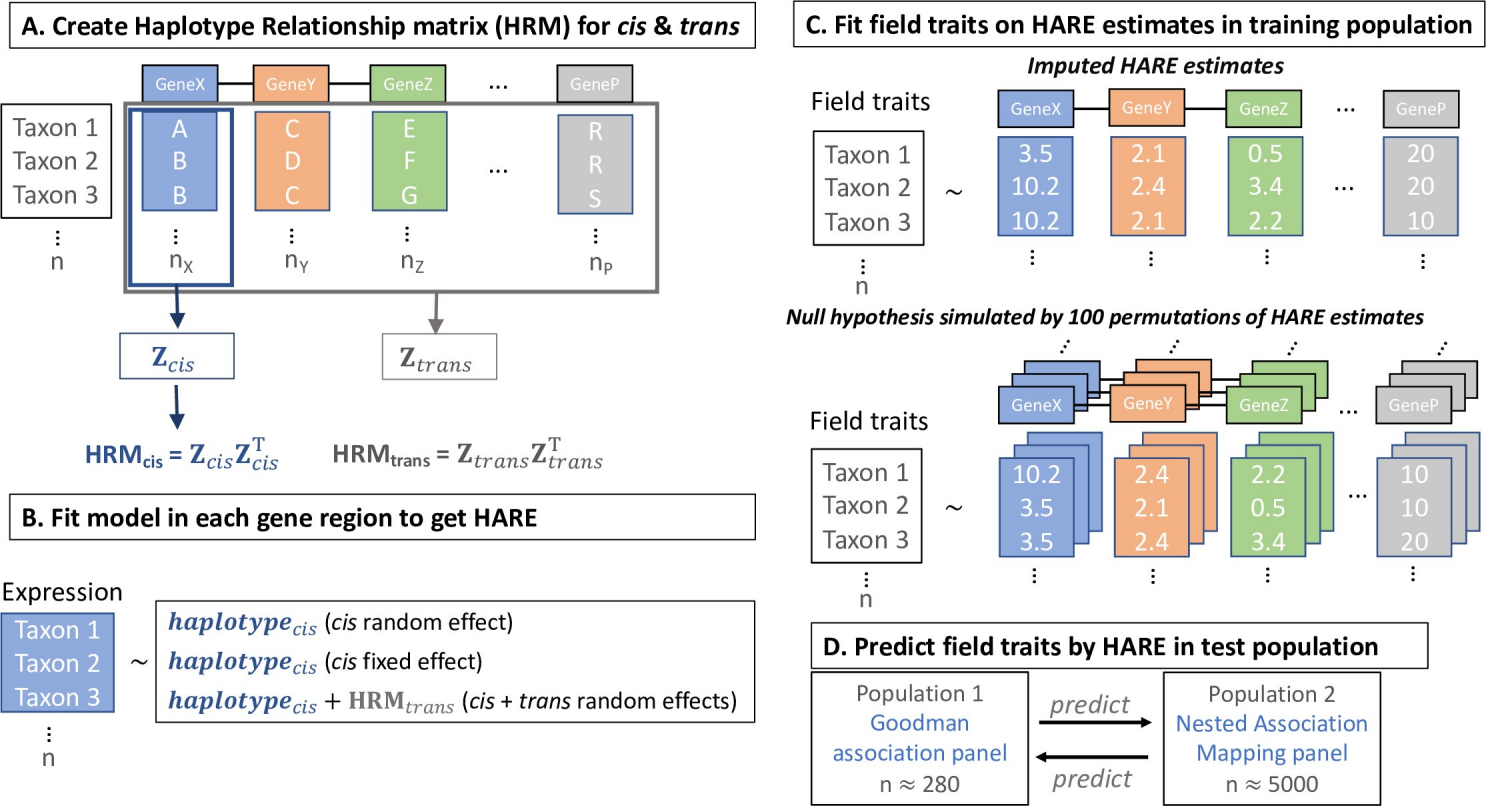
Experimental methods on calculating haplotype associated RNA expression (HARE) and using HARE to predict complex traits. A. The haplotypes of 26 NAM founders and one additional stiff stalk inbred line were identified in each gene region of the Goodman Association panel by mapping GBS reads (presented in detail in Valdes Franco et al. [27]) to the indexed pangenome of 27 lines. Haplotype relationship matrices (HRM_*cis*_) were created in each gene region and all genic HRM_*cis*_ were combined to get HRM_*trans*_ to control for *trans* effects, B. Using gene expression from 7 tissues [26], fixed or random effects models were fitted in each gene region with or without controlling for *trans* effects for each gene, C. Models were trained using field phenotypes in the Goodman panel or the NAM panel using HARE estimates or 100 randomly permuted HARE values while preserving haplotype structure, and D. Trained models were used to predict complex traits across populations.

**Fig 2:**
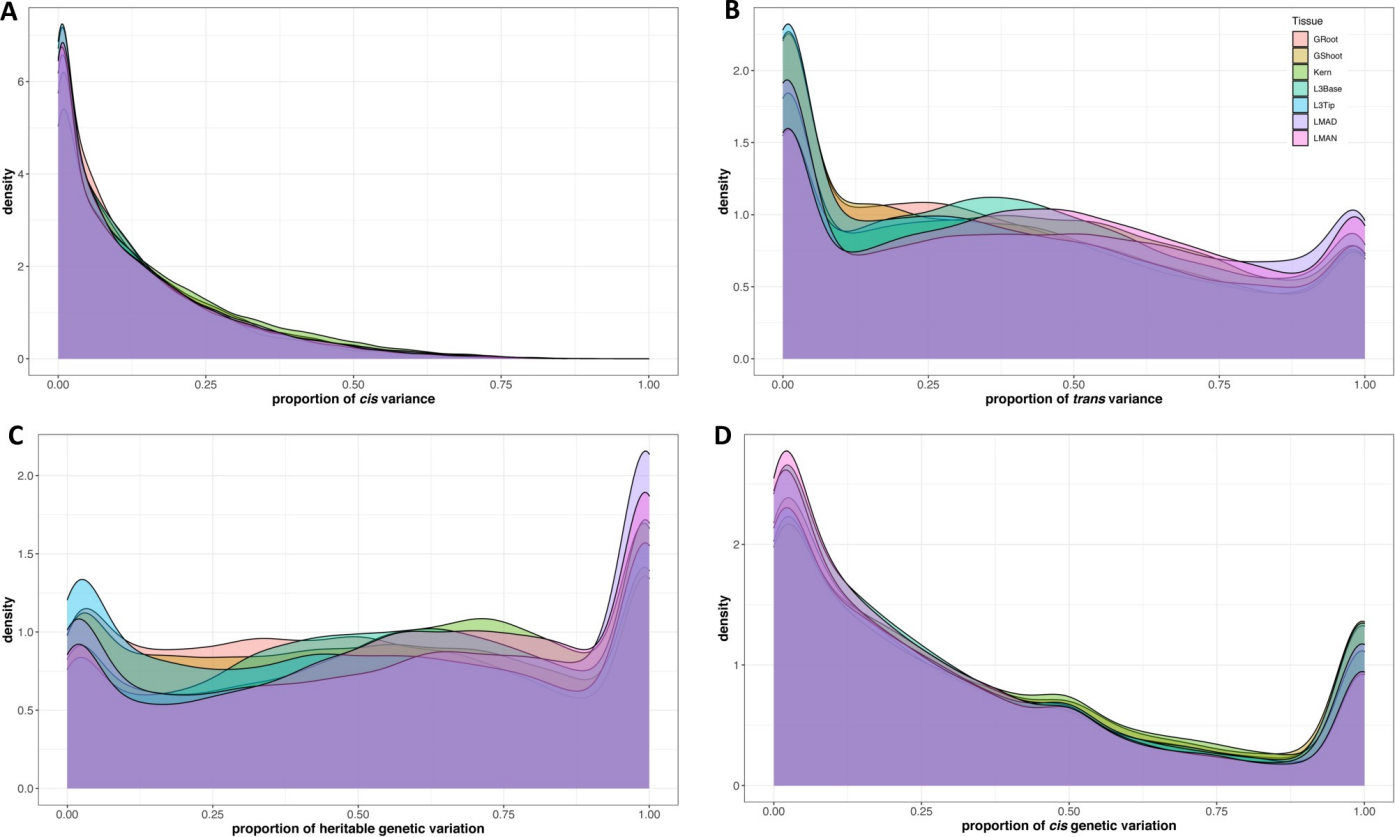
*Cis* haplotype explained one-third of the total genetic variance in expression. Proportion of variation explained by (A) *cis* and (B) *trans* components, (C) Proportion of heritable genetic variation over phenotypic variation and (D) proportion of heritable genetic variation explained by *cis* in gene expression across 7 different tissues. Heritable variation was calculated in each gene as the ratio of the sum of *cis* and *trans* variance to total variance. Different colors represent 7 diverse tissues in maize: germinating seedlings root (GRoot), germinating seedlings shoot (GShoot), 2 cm from the base of leaf 3 (L3Base), 2 cm from the tip of leaf 3 (L3Tip), mature mid-leaf tissue sampled during mid-day (LMAD), mature mid-leaf tissue sampled during mid-night (LMAN), and developing kernels harvested after 350 growing degree days after pollination (Kern).

We hypothesized that the majority of the expression would be contributed by *trans* acting factors as compared to the *cis* component. To test this hypothesis, we fit the model with haplotypes in each gene region as *cis* and the haplotype relationship matrix (HRM) combined across all genes as *trans* (model 3 and Fig 1B). For most of the genes, higher variance in RNA expression was explained by the *trans* component (Fig 2B) as compared to the *cis* component (Fig 2A), irrespective of the tissue. Overall, *cis* haplotypes contributed only 34% (31–38% across individual tissues) of the total genetic variation in expression across all genes.

The total heritability was quantified as the proportion of variation contributed by *cis* and *trans* components to the total variation in gene expression. Overall, the gene expression was highly heritable with an average ranging from 50% to 59% across tissues (Fig 2C). Though the gene expression was highly heritable, the heritability was primarily contributed by *trans* compared to *cis* (Fig 2D). The large effect of *trans* could be due to many small-effect molecular connections from *trans* regulators [11,28].

To test if the *cis* proportion of variation is different for highly expressed genes, we analyzed the result separately for a set of genes (~8,000 genes) with highest expression in each tissue. The *cis* haplotype explained a similar amount of variation (median 33%), however, the heritability increased slightly from a median of 54% to 60% across all tissues in a set of highly expressed genes.

### Transferability of Haplotype Associated RNA expression (HARE)

**Fig 3:**
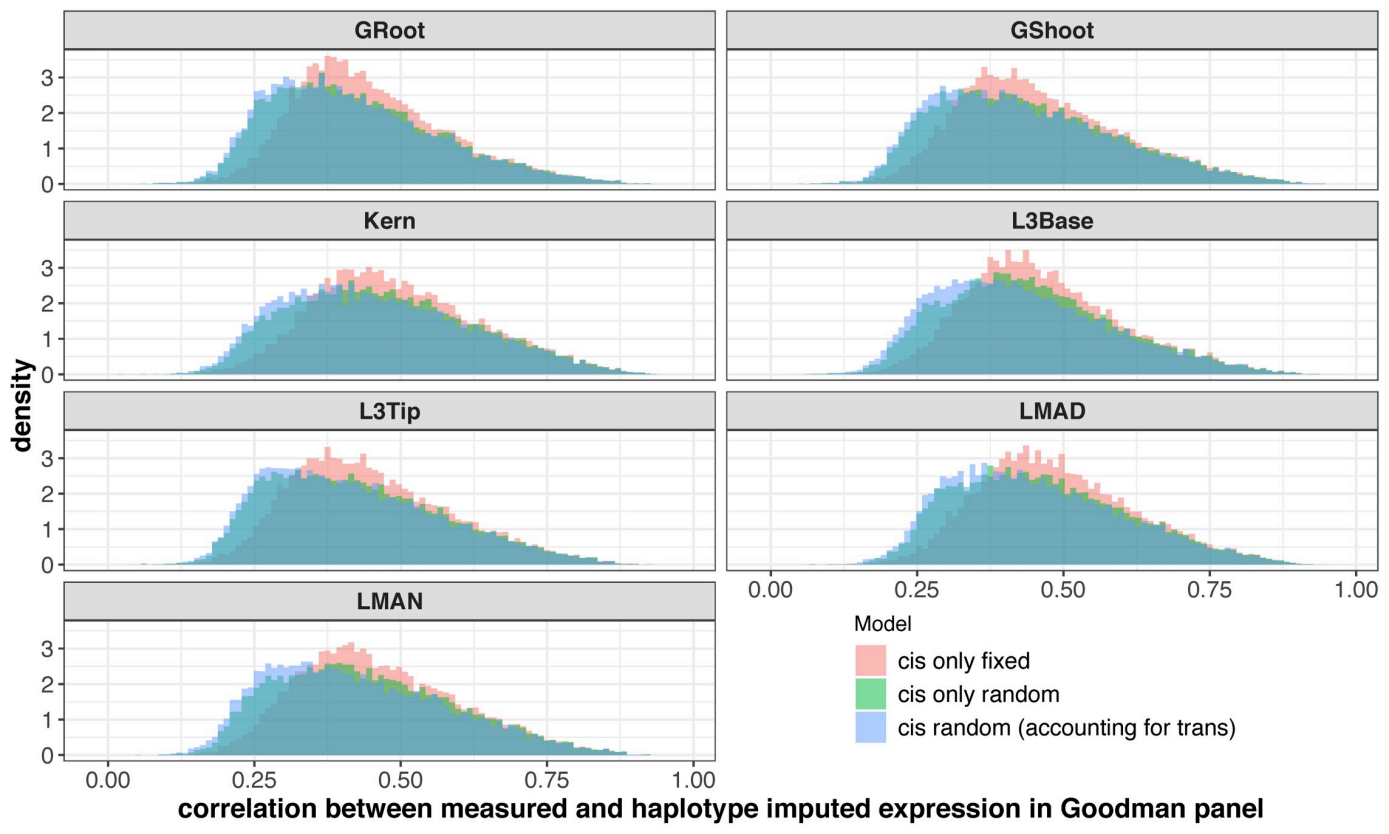
Haplotype associated RNA expression (HARE) was moderately correlated with measured RNA expression. The different colors represent HARE imputed from three statistical models: Model 1 (*cis* fixed effect), 2 (*cis* random effect), and 3 (*cis* + *trans* random effects) across 7 diverse tissues: germinating seedlings root (GRoot), germinating seedlings shoot (GShoot), 2 cm from the base of leaf 3 (L3Base), 2 cm from the tip of leaf 3 (L3Tip), mature mid-leaf tissue sampled during mid-day (LMAD), mature mid-leaf tissue sampled during mid-night (LMAN), and developing kernels harvested after 350 growing degree days after pollination (Kern). Transcripts from measured RNA expression was a result of genetic signals in both *trans* and *cis*. Therefore, the correlation was moderate for most of the genes.

We used three different models to estimate HARE. Model 1 and 2 included only *cis* effects fit either as a fixed or random, whereas model 3 included both *cis* and *trans* random effects. To determine how close the HARE estimates were to measured expression, we calculated the Pearson correlation coefficient between HARE estimates and measured expression levels across all genes in each tissues. HARE estimates describe the effect of a genic haplotype, so they capture only the *cis* component of variation in expression levels. Therefore, a correlation coefficient close to 1 implied that the majority of variation in gene expression was contributed by *cis*, whereas a correlation close to 0 meant most expression was contributed by *trans*. The overall distribution across all expressed genes was similar in all tissues and models with a mean correlation of 0.44, which indicated that a moderate amount of the variability in measured expression levels was captured by their *cis* component through HARE estimates (Fig 3).

**Fig 4:**
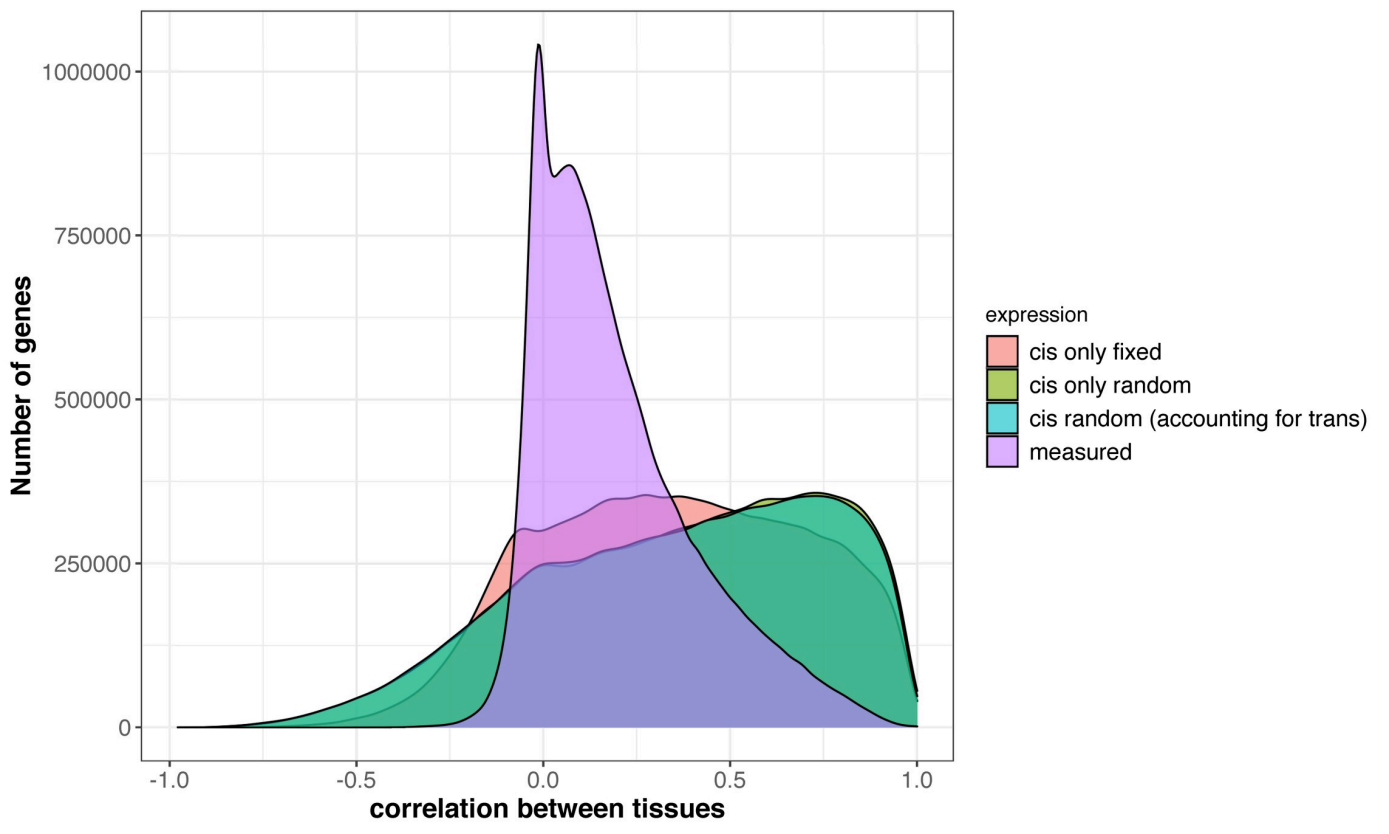
Haplotype associated RNA expression (HARE) was highly correlated across tissues as compared to measured transcript expression. Different colors represent HARE imputed from 3 statistical models: Model 1 (*cis* fixed effect), 2 (*cis* random effect), and 3 (*cis* + *trans* random effects), and measured transcript expression. The distribution is pairwise correlation of genes across 21 different combinations from 7 different tissues.

We hypothesized that HARE would include the transferable portion of gene expression by haplotype variation. To test this, we compared HARE and measured transcript expression for their correlation across tissues. Correlation coefficients ranged from -1 to 1 in all 21 different pairs of 7 diverse tissues (Figs 4 and S3). The median correlation coefficient was 0.14 across all tissue pairs in measured expression, whereas it was 0.4 in HARE. Correlation across tissues was larger for a set of highly expressed genes (~8,000 genes) as compared to the overall set. The median correlation across tissues increased from 0.14 to 0.21 in measured expression and 0.4 to 0.53 in HARE (S5 Fig) in the highly expressed gene set. HARE imputed from all three models followed similar trends of higher correlation for most of the genes across tissues than measured transcript expression. Closely related tissues, such as mature mid-leaf tissue sampled during midday (LMAD) and midnight (LMAN), were more correlated than other tissue combinations in both measured expression and HARE (S3 Fig), reflecting the influence of shared gene regulatory mechanisms driving these correlations.

To see if the large correlations between tissues were driven by genes with lower expression counts, we looked further into highly correlated and lowly correlated genes. We first divided genes into two sets: ‘highly correlated’ with correlations higher than 0.75, and ‘lowly correlated’ with correlations between -0.05 and 0.05 in all tissues. For both highly and lowly correlated sets, we looked at the proportion of genes with low counts (fragments per million counts <5). In mature leaf tissue expression, out of 10,600 genes with low correlation across tissues, only 3,000 genes had low expression counts, whereas, out of 11,500 genes with high correlation across tissues, only 1,400 genes had low expression counts. Therefore, genes with low expression (low fragment per million counts) did not drive the higher correlations of HARE estimates across tissues.

### Comparison between HARE models in genomic prediction

The high correlation of HARE estimates across tissues suggests that consistent and transferable genetic information is captured by HARE. The functional application of HARE was evaluated using genomic prediction within and across populations for 26 agronomically important traits in maize (Table 1). Transferability across populations was evaluated based on prediction accuracy, calculated as the Pearson correlation of observed and predicted trait values. The genomic prediction models were trained to predict traits within and across populations in maize using HARE estimates, random HARE estimates (randomly permuted values representing only haplotype structure; see Materials and methods), and pruned SNPs from HARE regions. First, we compared prediction accuracies in three sample traits (days to anthesis, days to silking, and plant height) using HARE estimates and random HARE from three different methods (models 1, 2, and 3). We did not see any significant differences in accuracy using any of these imputation methods (S6 Fig), so we used HARE estimates from model 3 (*cis* effects adjusted from *trans* effects) to predict all 26 traits within the Goodman Association panel and across panels in the Goodman Association and NAM panel. Additionally, HARE resulted in similar prediction accuracy compared to pruned SNPs from the HARE regions (S9 and S11 Figs) in both populations. The similar accuracy by HARE compared to SNPs was achieved in spite of ~10 times fewer variables in HARE models (see Material and methods).

**Table 1:** Selected traits for genomic prediction.

Category	Traits	Reference
**Flowering**	Days to silking	[25]
	Days to anthesis	[25]
	Anthesis silking interval	[25]
	Tassel length	[22]
	Tassel primary branches	[22]
**Morphology**	Plant height	[25]
	Ear height	[25]
	Leaf length	[22]
	Leaf width	[22]
	Leaf angle	[22]
	Nodes below ear	[22]
	Nodes above ear	[22]
	Number of brace roots	[22]
**Yield related**	Cob diameter	[22]
	Cob length	[22]
	Ear row number	[22]
	Kernel number per row	[22]
	Ear mass	[22]
	Cob mass	[22]
	Kernel wt	[22]
	Test wt	[22]
	Total kernel number	[22]
**Kernel composition**	Starch	[24]
	Protein	[24]
	Oil	[24]
**Disease**	Southern leaf blight	[23]

### Within-panel prediction by HARE, compared to measured expression and haplotype structure (random HARE)

**Fig 5:**
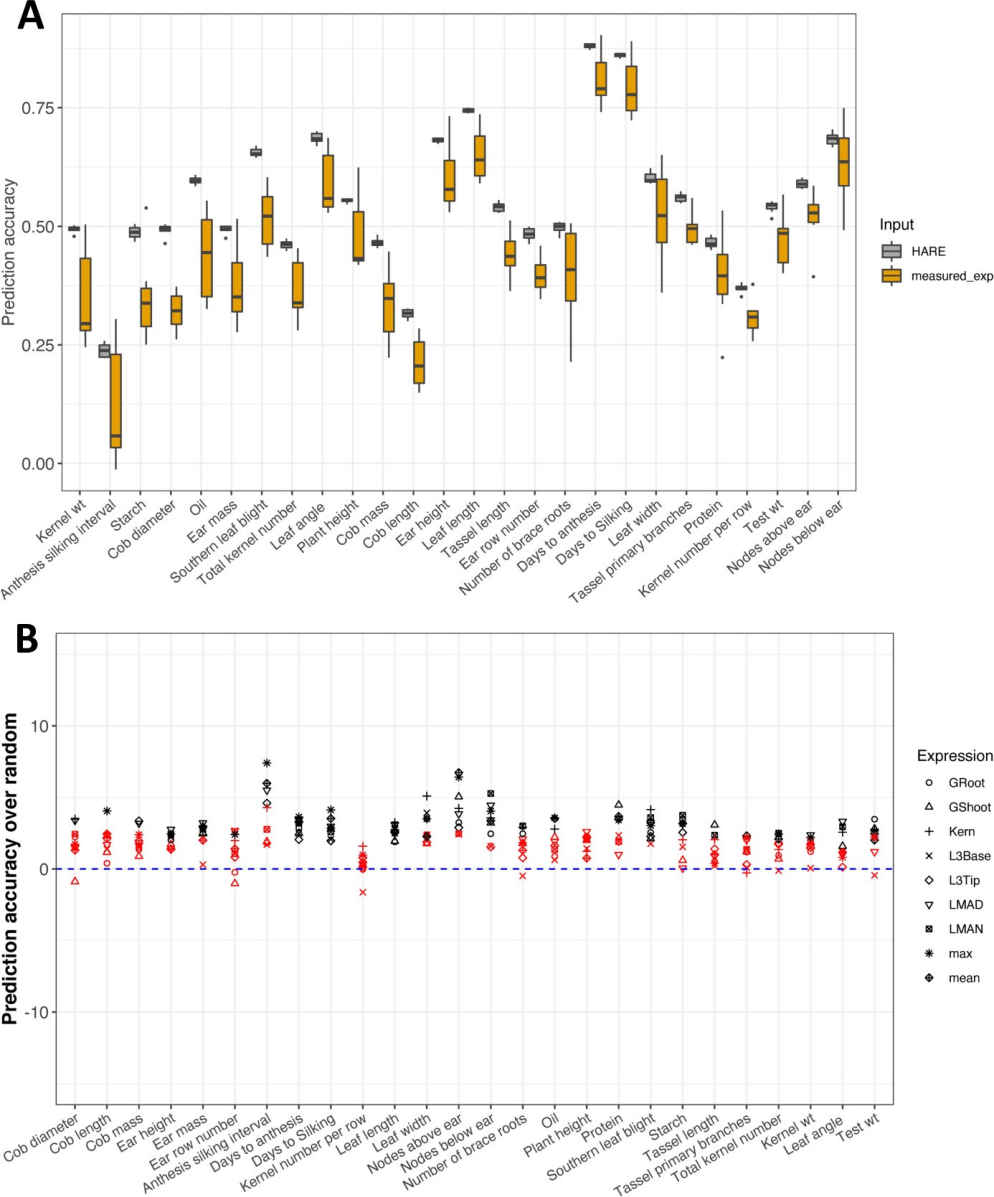
HARE improved within-panel prediction accuracy over measured expression and random HARE for most of the traits. (A) Prediction accuracy within the Goodman Association panel using HARE and measured expression (measured_exp) from all tissues arranged by prediction differential. (B) Change in prediction accuracy using HARE over the mean accuracy from random HARE (blue dashed line). Different symbols represent HARE from different tissues. The black shapes represent statistically significant differences at P-value <0.05 and red shapes are without significant differences. P-values calculated using Monte Carlo procedure.

The comparison of prediction accuracy using measured expression and HARE was conducted in the Goodman panel to test the hypothesis that predictions by HARE would be more accurate than those by measured expression. Prediction accuracy using measured expression was highly variable across traits and tissues; in contrast, prediction accuracy by HARE was less variable across tissues. The highest accuracy was observed for flowering time traits (e.g., days to anthesis up to 0.9), using HARE from all tissues, or measured expression from mature mid-leaf tissues (LMAD and LMAN), and pruned SNPs from the HARE regions. Overall, HARE resulted in higher prediction accuracy for *all* 26 traits, compared to measured expression in any tissue (Figs 5A and S7). The highest accuracy increase was for the number of brace roots, which increased from 0.21 to 0.5 using HARE from germinating root (S7 Fig). However, the median increase across tissues was highest for trait kernel weight, which increased from 0.27 to 0.5 (Fig 5A). HARE also resulted in significantly higher prediction accuracy compared to haplotype structure only (random HARE) for 24 traits (P-value < 0.05) (Fig 5B). Additionally, the median prediction accuracy using HARE was slightly higher in 15 traits when compared with SNPs (S9 Fig). Therefore, partitioning expression at the level of gene haplotypes results in higher prediction accuracy, when compared to predictions by measured expression or haplotype structure (random HARE).

### Cross-panel prediction using HARE as compared to haplotype structure (random HARE)

**Fig 6:**
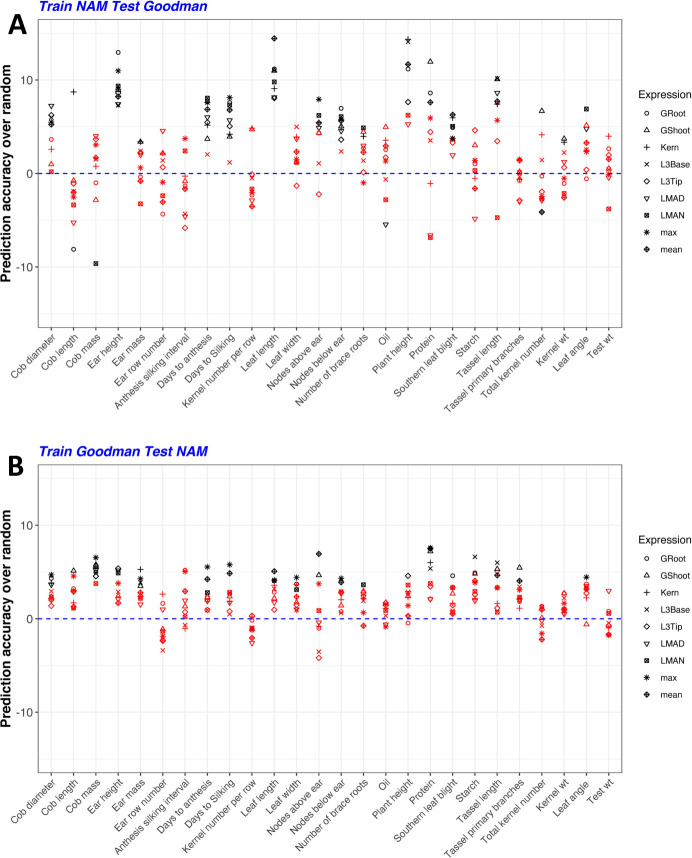
HARE improved cross-panel prediction accuracy over random expression values for most of the traits. Change in prediction accuracy using HARE over the mean accuracy from random HARE (blue dashed line) for models (A) trained in NAM and tested in the Goodman panel, (B) trained in the Goodman panel and tested in NAM. The different symbols represent HARE from different tissues: germinating seedlings root (GRoot), germinating seedlings shoot (GShoot), 2 cm from the base of leaf 3 (L3Base), 2 cm from the tip of leaf 3 (L3Tip), mature mid-leaf tissue sampled during mid-day (LMAD), mature mid-leaf tissue sampled during mid-night (LMAN), and developing kernels harvested after 350 growing degree days after pollination (Kern). The black shapes represent statistically significant differences at P-values <0.05 and red shapes represent no statistical significance. P-values were calculated using a Monte Carlo procedure.

**Fig 7:**
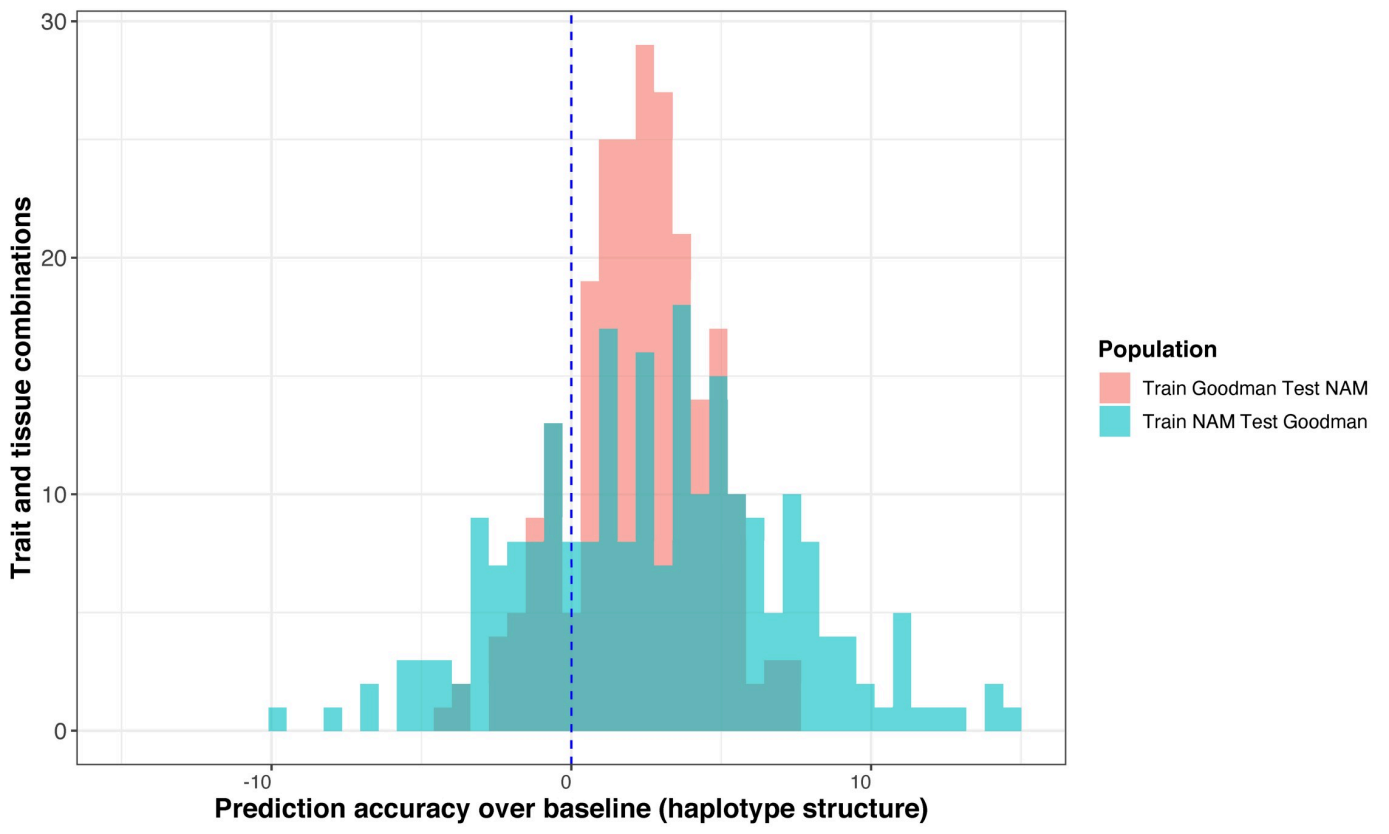
HARE increased prediction accuracy by up to 14% when the model was trained in NAM and tested in the Goodman panel. Summarized differences in prediction accuracy using HARE over the mean accuracy from random HARE (representing haplotype structure) across 26 phenotypes and 7 diverse tissues. The blue dashed line is the mean prediction accuracy using random HARE across each trait and tissue combination.

For all 26 traits and 7 diverse tissues, models were trained using HARE or random HARE in cross-panel prediction (from NAM to the Goodman Association panel and *vice versa*) to determine if HARE carried functional information beyond haplotype structure across populations. HARE often improved prediction accuracy of many traits when the model was trained in NAM or the Goodman panel as compared to random HARE. HARE significantly increased accuracy 34.6% of the time across trait and tissue combinations when the model was trained in NAM (Fig 6A and S1 Table) and 21.8% of the time when trained in the Goodman panel (Fig 6B and S2 Table). Out of 26 traits, the accuracy was significantly higher in 17 traits when trained in NAM and tested in the Goodman panel, *versus* 19 traits when trained in the Goodman panel and tested in NAM (P-value < 0.05). However, the increase in accuracy was highly variable across these two panels. The increase was as high as 15% (for morphological traits: plant height and leaf length) when the model was trained in NAM and tested in the Goodman panel; whereas it was less than 10% when the model was trained in the Goodman panel and tested in NAM (Figs 6A, 6B and 7). The difference in prediction accuracy was also observed with pruned SNPs from HARE regions across the two populations (S10 Fig).

The increase in accuracy over random HARE was also observed when a model was trained only in the sample of 250 NAM RILs (a similar size as the Goodman panel, 10 random RILs from 25 families) to predict 3 traits (days to anthesis, days to silking, and plant height) in Goodman panel (S8 Fig). When the model was trained in NAM, the increase in prediction accuracy reached up to 16% over random HARE for the morphological traits, 10% for flowering traits, 8% for yield traits, 12% for kernel composition, and 6% for disease related traits (S1 Table). In general, traits in yield and disease-related categories had the lowest accuracy when compared to the traits in other categories using HARE or pruned SNPs from HARE regions in both populations (S11 Fig and S1 Table). Genomic prediction models using HARE could improve prediction accuracy with simple computational work without any additional cost for data generation. Therefore, haplotype-based models can improve genomic prediction across populations; however, the improvement depends on the traits of interest. The overall number of significant improvements was higher when using mean or maximum expression as inputs rather than individual tissue expression. Therefore, integrating expression from diverse tissues (e.g., mean or maximum expression) may further improve prediction accuracy.

## Discussion

### Cis haplotypes explained one-third of the genetic variation in expression

Consistent with other studies, we found the vast majority of expression to be heritable [28–30]. In eQTL mapping, *cis*-eQTL can seem predominant as they are frequently the single largest QTL for a given gene, but this is likely a power and multiple testing issue [12]. By using variance partitioning and assuming a polygenic model, we are likely accurately estimating the relative importance of these two components, *cis* and *trans*.

We showed that the *cis* haplotype explained around 34% of variation in expression, which given the relatively small size of *cis* region and rapid linkage disequilibrium decay surrounding the gene, is a strongly enriched proportion of variance explained by *cis*. Similar results were observed by Lemmon et al. [30] in maize and teosinte using hybrid allele-specific expression, a complementary technique to our approach. These allele-specific results and our study agree with biological knowledge, where dozens of transcription factors likely regulate each gene [13]. These transcription factors are a result of any regulatory genes modelled as *trans*. In contrast, *cis* variability is a results from variation within or around single gene, empirically lowering the variability explained by a *cis* when compared to the overall *trans* effect, as observed in similar experiments in human and yeast [12,29].

### HARE was highly transferable across tissues as compared to measured transcript expression

Variation in gene expression across tissues, developmental stages, genotypes, and experimental conditions has been shown in earlier studies in plants and humans [26,31–33]. Low correlations in expression for similar genes have been observed across populations in Mogil et al. [34]. Therefore, high gene expression in one population may not always be as high in diverse panels. The lack of strong correlations in measured transcript expression may result from *trans* effects in gene expression specific to tissues, genetic backgrounds, or environmental conditions [11] (S4 Fig). With HARE, we observed higher transferability across tissues as this portion of the variation in gene expression was less sensitive to environmental perturbations and biological contexts. The *cis* regulatory mutations affect expression of fewer genes than *trans* effects, resulting in less pleiotropy and fewer functional tradeoffs [35]. In the absence of large pleiotropic effects, selection can act more consistently, so *cis* effects may be more transferable across different backgrounds [35,36].

HARE can integrate a rich allelic series that is more transferable across different contexts than measured transcript expression which is a result of *cis*, *trans*, their interaction, and environmental effects. Allelic richness is more pronounced in species like maize, which has 20 times higher nucleotide diversity than human beings [37]. Because of high allelic richness in maize, a wide range of possible alleles might lead to the same molecular outcome (for example, gene expression, protein expression), a concept known as equifinality. Due to equifinality, it has been observed that allelic variants are not always shared across genomes, and transcription is not always correlated with translation [38]. However, the *cis* portion of expression that summarizes allelic richness is highly transferable across tissues. The effect of *cis* variants in HARE regions located in the close promoter, 5’ and 3’ untranslated regions, introns and the gene regions are likely consistent across tissues. Further research is needed to understand the effect of enhancer and tissue interactions in the variation on *cis* effects, however the current study suggests that it is not the dominant factor.

### HARE improved prediction over measured expression

Biological information flows along the central dogma from the genome to the transcriptome, proteome, metabolome, and finally to complex phenotypes [39]. For most trait and tissue combinations, transcriptome expression yielded lower prediction accuracy when compared to HARE (Figs 5A and S7). Furthermore, we observed less variability in prediction accuracy using HARE, which points to the context-dependence of RNA expression. HARE owes its consistent advantage in prediction accuracy to functional information that does not include non-genetic sources of variability in RNA expression (interactions among *trans* and *cis* factors and environment). Transcriptome data from mature leaf tissues yielded higher prediction accuracy for most of the phenotypes, compared to the young developing tissues from shoots or roots, and kernel tissue [26]. Therefore, gene expression in different tissues may not capture the same functional information. Most of the phenotypes in this study were measured under field conditions in mature tissues or kernels in different seasons (e.g., flowering traits, agronomic traits). Therefore, mature leaf tissue expression measured in the field should be “closer” to these phenotypes, allowing higher prediction accuracy than expression at the seedling stage measured under controlled conditions. With HARE, the contextual issue was less pronounced, resulting in more stable prediction accuracy from any of these tissues.

### A baseline for comparison of genomic prediction models is important

To determine if HARE carries functional information in addition to haplotype structure in genomic prediction, we used genetic signals produced by random HARE as a baseline for our genomic prediction models. Prior studies have used different baselines to assess the predictive ability of their genomic prediction models. For example, Westwhues et al. [40] used traditional pedigree BLUP as a baseline to compare the predictive abilities of genomic sequences, metabolomes, transcriptome or a combinations of these; Azodi et al. [2] used the first five principal components in the marker data and compared them with the genomic and transcriptome data; and Li et al. [5] used a genomic BLUP model with SNP data and compared it with the integration of additional endophenotypes. In this study, randomly permuting the HARE estimates while preserving haplotype structure allowed us to assess the accuracy of genomic prediction in the absence of functional information in haplotype values and test the significance of HARE over haplotype structure directly. The significantly higher prediction accuracy of HARE affirmed that HARE carried functional information beyond haplotype structure.

### HARE captured functional information beyond haplotype structure

The benefits of using haplotypes and transcriptomes over SNPs in genomic prediction has been highlighted in earlier studies [4,5,7–10]. Our study here integrated both haplotypes and transcriptome information (as HARE) in the prediction of complex traits. Haplotypes can capture epistasis in genic regions, which cannot be captured by additive SNP effects [4–6,40]. Another issue in genomic prediction models is overparameterization, where there are more predictors than observations [2,41]. By using transcriptome data rather than SNPs as predictors, the feature dimension can be reduced from millions to thousands, making the model more transferable by addressing the curse of dimensionality. Even though we did not see large improvement in genomic prediction accuracy for HARE over SNPs, HARE captures functional information through imputed gene expression, and concisely summarizes genetic variation with ~10 times less variables than pruned SNPs. Critically, HARE may also enable cross-population prediction even when few polymorphisms are shared across populations. In contrast, prediction based on SNPs require that polymorphisms at each SNP to be shared across populations.

With HARE, we have effectively captured transcription, but not translation. Genome-wide prediction needs to model protein abundance and three-dimensional structural changes to fully understand their biological impact on phenotype. Tools like AphaFold2 are likely to help make haplotype imputation of protein structure tenable. Our approach could be improved by functional annotations about gene expression and protein structure prediction, and prior information about their effects on phenotype, which could weight the importance of genes in HARE.

### Tapping into a new source of functional information using HARE

**Fig 8:**
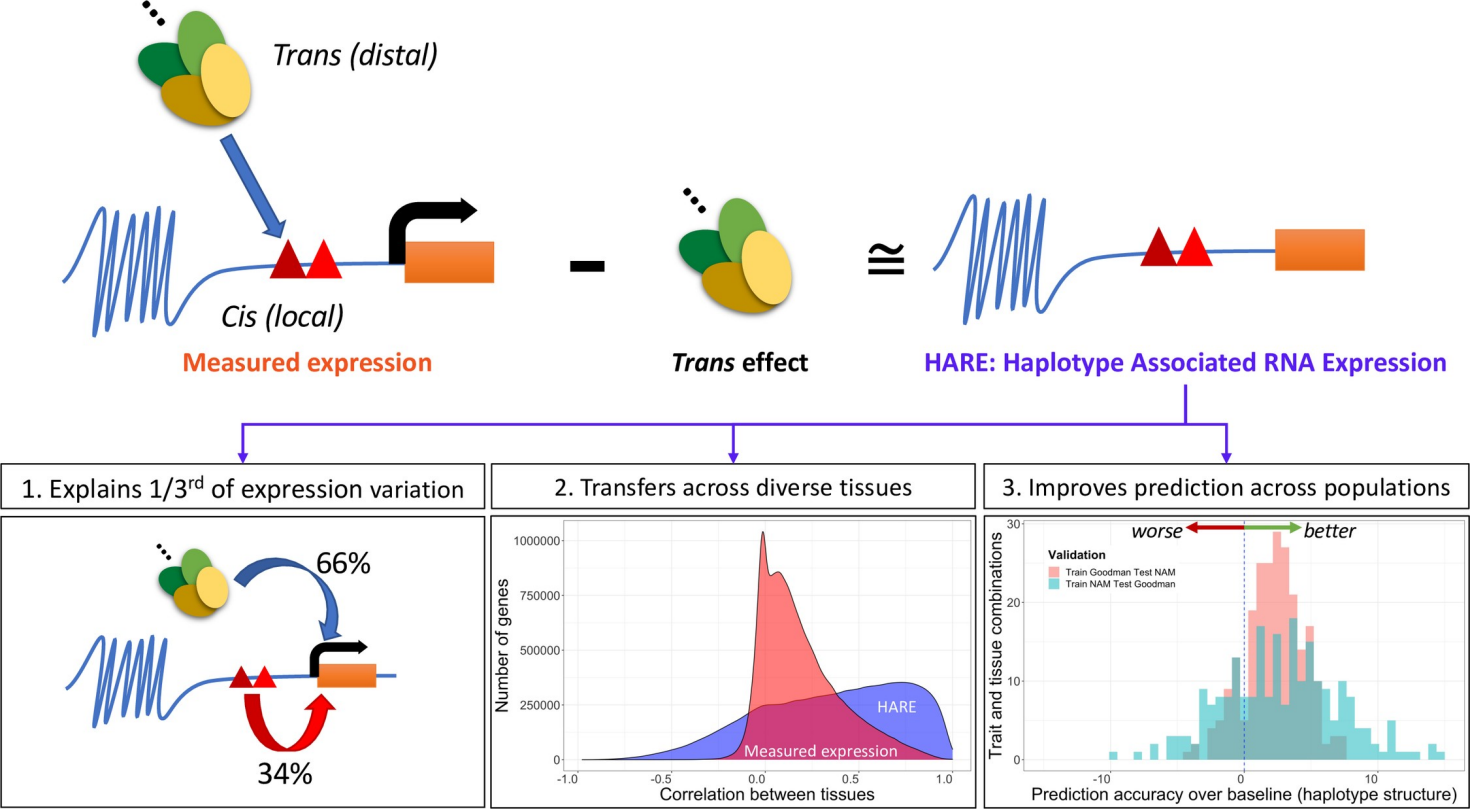
Graphical Summary of the Study. *Cis* haplotype associated RNA expression (HARE) was obtained from subtracting *trans* effects from measured expression. 1. The *cis* haplotypes explained one-third of the variation in expression. 2. The HARE estimates were highly transferable across tissues compared to measured expression. 3. HARE improved prediction within and across populations in maize.

Here, we presented a novel method for imputing HARE using the Practical Haplotype Graph (PHG) and a mixed model approach (Figs 1 and 8). Measuring the transcriptome in multiple tissues for every population is expensive, while imputing expression is more accessible and cost effective. Here we used existing transcriptomes profiled in 7 diverse tissues of the Goodman Association panel consisting of 280 diverse lines and assemblies of subsets of lines to get HARE estimates. HARE estimates were then imputed in the NAM panel consisting of 5000 lines using the PHG. Imputing expression was not only cheaper, it also contributed to more robust genomic predictions as compared to random tagging of haplotypes. Other methods, based on deep learning techniques, for predicting expression from genomic sequences were previously reported, however, these methods were trained on few genomes, not on population data [15,42]. Our approach requires sparse sequencing data to obtain haplotypes from the PHG, and expression in some genotypes. Therefore, it is less computationally intensive and more cost-effective than approaches based on deep neural networks applied to genomic sequences.

## Conclusion

We showed that by leveraging the diverse high-quality assemblies through a haplotype graph, we can impute *cis* Haplotype Associated RNA Expression in diverse panels. By showing higher transferability across tissues and moderate correlation with measured expression, we have demonstrated that imputing HARE could generate more stable gene expression measurements across biological contexts. The important consideration in many genomic prediction and transcriptome studies is the cost of generating the genomics and transcriptomics data. Our approach here utilizes sparse sequencing data to obtain haplotypes and impute expression on those haplotypes using previously generated gene expression data measured in related genotypes.

Also, we have demonstrated that HARE estimates could improve genomic prediction for most complex traits in maize over haplotype structure or measured expression. Although HARE did not outperform SNPs in our validations, HARE has important advantages for cross-panel prediction: it represented genomic variation more succinctly (with ~10 times fewer variables than pruned SNPs) and it captured explicit functional information through imputed gene expression. With HARE, we have effectively addressed modeling RNA expression between tissues and diverse genotypes, however, we have not addressed translation mechanisms or low-frequency coding variants that may affect the translation process or final protein structure. Refining expression estimates and understanding how coding variants impact protein structure and function are crucial in modeling how information flows along the central dogma of biology to impact phenotypic variation in maize and other crops.

## Materials and methods

### Phenotypic data

Two maize panels were evaluated for prediction accuracy: the US Nested Association Mapping (NAM) panel and the Goodman Association panel representing the genetic diversity among maize elite inbred lines. The NAM panel was developed from 25 parents crossed to a common parent B73 and selfed to obtain 200 homozygous recombinant inbred lines (RILs) from each cross, as described in McMullen et al. [20] and Gage et al. [21]. The Goodman Association panel represents the global diversity of inbred lines in public maize breeding programs, including ~280 genotypes from tropical and temperate regions, sweet corn, and popcorn lines [19]. The 25 NAM founders are part of the Goodman Association panel, so we excluded them from the Goodman Association panel set for cross-panel prediction.

We evaluated genomic prediction models for 26 traits belonging to different groups: flowering, morphology, yield-related, kernel composition, and disease (Table 1). These traits were chosen from 4 publications where they were jointly phenotyped in the two panels [22–25]. Phenotypic evaluations for these traits were performed in 2006 and 2007 across 11 environments, though not all traits were measured in all environments. The field experiments were conducted using an incomplete block alpha lattice design. The phenotypic values were best linear unbiased predictors (BLUPs). Details on the phenotypic measurement and BLUP calculation are presented in the respective studies (Table 1).

### PHG database for NAM and Goodman Association panels

Details on the Practical Haplotype Graph (PHG) were described in Valdes Franco et al. [27]. In brief, the database consisted of the genomes of 26 NAM parents and one additional stiff stalk inbred B104. The genomes were divided into reference ranges, where the edges of each reference range were defined by gene boundaries in B73 RefGen_v5. A total of 71,354 reference ranges were identified, where half of them were genic regions. The genotyping-by-sequencing (GBS) reads from NAM RILs [43], and the Goodman Association panel [44] were mapped to the PHG database to identify the haplotypes in these populations based on the 27 genomes in the PHG. The SNP calls thus generated were tested for error rate and heterozygosity, imputation accuracy as presented in the original publication [27].

### Haplotype ID analysis

For each line in the NAM and Goodman panels, a haplotype ID was obtained in each reference region from the PHG database using function pathsForMethod in the rPHG package in R (Bradbury et al., in prep). Since the reference ranges included both genic and intergenic regions, the ranges were filtered to obtain only the genic reference ranges based on the B73 RefGen_v5 annotations. To assess the diversity or information content of haplotypes, we calculated the Shannon entropy of haplotype’s frequency in genic reference ranges using the maximum likelihood method in the R entropy package [45]. Since the NAM population has low genetic diversity because of the over-representation of the common parent B73 haplotypes at a high frequency, NAM should be associated with a lower haplotype entropy than the highly diverse Goodman Association panel.

SNPs data were imputed from the haplotypes for each line in the NAM and Goodman Association panels from the PHG database. The SNPs in the same reference regions as HARE were filtered for minor allele frequency higher than 0.05 and major allele frequency smaller than 0.95, using TASSEL Version 5.0 [46] resulting in ~800K SNPs. The SNPs were then LD filtered to remove SNPs with pairwise R2 > 0.9 within 100 kb windows, using SNPRelate package in R [47] resulting in 156,222 pruned SNPs.

### Gene expression data

Gene expression data was obtained from Kremling et al. [26]. Details on sampling and expression quantification are presented in the original publication. 7 different tissues (germinating seedlings root: GRoot; germinating seedlings shoot: GShoot; two centimeters from the base of leaf 3: L3Base; two centimeters from the tip of leaf 3: L3Tip; mature mid-leaf tissue sampled during mid-day: LMAD; mature mid-leaf tissue sampled during mid-night: LMAN; and developing kernels harvested after 350 GDD after pollination: Kern) were included in the analysis. Using the expression from 7 different tissues, maximum expression, and mean expression per gene was calculated using a custom script in R.

The gene expression data was uplifted from B73 v3 to B73 v5 by mapping B73 v3 genes to the B73 v5 reference genome. Genes that did not map or mapped in multiple positions were removed from the analysis. The final genic haplotype matrix included 18,004 genes with one-to-one correspondence between the two genome versions.

### Variance partition in gene expression

The variance components in gene expression were estimated using the R package regress and genetic values were obtained by solving mixed model equations by restricted maximum likelihood (REML) [48]. We fit a linear mixed model for each gene to partition variance into the fraction attributable to the genic reference range (haplotypes representing *cis* effects) with and without controlling for *trans* effects. The effects of haplotypes in the genic reference range were fit as fixed or random as described below. The statistical models for variance partition were:

$$1.\;y=1\mu +Z_{cis}\alpha +e\;(cis\;fixed\;effects)$$

$$2.\;y=1\mu +u_{cis}+e\;(cis\;random\;effects)$$

$$3.\;y=1\mu +u_{cis}+u_{trans}+e\;(cis+trans\;random\;effects)$$

where **y** is the RNA expression at a given gene, **Z**_*cis*_ is the design matrix for the gene’s *cis* haplotypes, **α** is the vector of fixed effects of *cis* haplotypes on gene expression, $u_{cis}∼N(0,H_{cis}\sigma_{cis}^{2})$ is the vector of *cis* haplotypic effects ($H_{cis}=\frac{Z_{cis}Z_{cis}^{T}}{tr(Z_{cis}Z_{cis}^{T})/n}),\;u_{trans}∼N(0,H_{trans}\sigma_{trans}^{2})$ is the vector of *trans* haplotypic effects as captured by the design matrix **Z**_*trans*_ for haplotypes at all genes $\left(H_{trans}=\frac{Z_{trans}Z_{trans}^{T}}{tr(Z_{trans}Z_{trans}^{T})/n}),e∼N(0,I\sigma_{e}^{2}\right)$ is the vector of errors, *n* is the number of lines in the panel(s), and *tr* is the trace operator (sum of diagonal elements).

The proportion of variance explained by *cis* and *trans* components was estimated from model 3. The *cis* haplotype heritability was estimated as $h_{cis}^{2}=\frac{\sigma_{cis}^{2}}{\left(\sigma_{cis}^{2}+\sigma_{trans}^{2}+\sigma_{e}^{2}\right)}$, and *trans* heritability was estimated as $h_{trans}^{2}=\frac{\sigma_{trans}^{2}}{\left(\sigma_{cis}^{2}+\sigma_{trans}^{2}+\sigma_{e}^{2}\right)}$. The proportion of heritable variance is the total proportion of variance explained by *cis* and *trans* estimated as $\frac{\sigma_{cis}^{2}+\sigma_{trans}^{2}}{\left(\sigma_{cis}^{2}+\sigma_{trans}^{2}+\sigma_{e}^{2}\right)}$, and *cis* portion of heritable variance was estimated as $\frac{\sigma_{cis}^{2}}{\left(\sigma_{cis}^{2}+\sigma_{trans}^{2}\right)}$.

### Haplotype associated RNA expression (HARE)

The HARE estimates were obtained using the regress package in R and genetic values were obtained by solving mixed model equations by REML [48]. Models 1, 2, and 3 were used to obtain HARE estimates for each haplotype in all genic regions.

Expression matrices were generated for genes in the Goodman panel and NAM based on the 27 haplotypes from NAM parents and B104. Missing haplotype expression was imputed using mean imputation using a custom script in R. The HARE expression matrix was compared with the measured expression matrix in the Goodman panel by pairwise correlation of genes using the *cor* function in R. Pairwise correlation was calculated between measured expression and HARE estimated across all genes. Similarly, transferability across tissues were assessed by pairwise correlation of genes across all 21 different combinations of 7 tissues for both measured expression and HARE.

### Genomic prediction model and model performance

The genomic prediction model was fit using ridge regression [49] using the glmnet package in R [50].

For a given set of *n* individuals and *p* genes, the following linear model was fit:

y=Xβ+ε

Where **y** is a *n*-vector of phenotypic values, **X** is the *n* x *p* matrix of genomic features: measured expression (*p* = 18,004), HARE estimates (*p* = 18,004), or SNPs (*p* = 152,600). **ε** is the vector of errors, where $\varepsilon ∼N(0,I\sigma_{\varepsilon }^{2})$, and **β** is the *p*-vector of effects of features on phenotypes, estimated by $\hat{\beta }={argmin}_{\beta }\{{\left\|y-X\beta \right\|}_{2}^{2}+\lambda {\left\|\beta \right\|}_{2}^{2}\}$ where ${\left\|.\right\|}_{2}^{2}$ is the squared *l*_2_-norm (the sum of squared elements of a vector) [51,52]. The optimal value of the regularization parameter λ was determined by minimum mean squared error in 10-fold cross-validation in the glmnet package.

### Assessment of genomic prediction ability

Part of the signal in genomic prediction by HARE may have been due to the sharing of haplotypes in these populations. Therefore, we established a baseline for genomic prediction by using random HARE estimates for haplotypes while preserving the haplotype structure (random HARE). For random HARE, the HARE estimates were permuted at the haplotype level, so that each gene had the same HARE estimates, but HARE estimates were randomly matched to haplotypes. For example, if lines 1 and 2 carried the same haplotype at gene *j*, after permutation, both lines got the same random value for imputed expression (Fig 1). The significance of using HARE over random HARE was assessed by using a Monte Carlo procedure with 100 random permutations of HARE [53]. P-values are calculated as $\frac{\left(r+1\right)}{\left(k+1\right)}$ where *k* = 100 is the total number of permutations and *r* is the number of permutations with accuracy greater than using HARE (accuracy of random HARE greater than accuracy of HARE).

The prediction accuracy of the genomic prediction models was defined as the Pearson correlation coefficient between the observed trait values (**y**) and predicted values ($\hat{y}$) in each of the test sets using the *cor* function in R.

### Within-panel prediction

In within-panel prediction, the prediction was carried out only in the Goodman panel using the measured expression, HARE, 100 random HARE (representing haplotype structure), and pruned SNPs from HARE regions. We used a repeated random sub-sampling validation (Monte Carlo cross-validation) for all data sets. For that the panel was randomly partitioned into 80% training set and 20% testing set and partitions were repeated 20 times. Pearson correlation was individually calculated in each of the 20 partitions and averaged over partitions to test for significance. For a single trait and tissue combination, the model was run for 2000 times for random HARE, and 20 times for HARE, measured expression, and SNPs.

### Cross-panel prediction

For cross-population prediction, the model was either trained in NAM and tested in the Goodman panel or vice versa for all traits, using HARE estimates, 100 random HARE estimates, or SNPs from HARE regions. The NAM founders are part of the Goodman Association panel, so we excluded those lines from the Goodman Association panel for cross-panel prediction. To account for sample size differences (5000 in NAM *versus* 250 in the Goodman panel), 20 random subsets of NAM equivalent to the size of Goodman panel were created, taking 10 RILs from each family. The model was trained in the 20 random subsets of NAM RILs and predicted in the Goodman panel and vice versa for three sample traits: days to anthesis (DTA), days to silking (DTS), and plant height (PH). The prediction accuracy was averaged across 20 random subsets.

## Supporting information

S1 Fig Phenotypic distribution of 26 traits in NAM and the Goodman Association panel. (TIF)

S2 Fig Haplotype frequency distribution in the (a) Goodman Association panel and (b) NAM panel across all genic reference ranges. Haplotype entropy in (c) Goodman and (d) NAM panel in each reference range. Median haplotype frequency were 8 and 100 in the Goodman and NAM, respectively, resulting in higher entropy in the Goodman panel as compared to NAM. Entropy was calculated from haplotype frequency in each reference region.
(TIF)

S3 Fig Correlation distribution of expression between tissues. The four panels represent HARE estimates from models 1 (*cis* only fixed), 2 (*cis* only random), and 3 (*cis* random while accounting for *trans*), as well as measured expression. The different color lines in each panel represent 21 different combinations of the 7 different tissues as labeled on the right: germinating seedlings root (GRoot), germinating seedlings shoot (GShoot), 2 cm from the base of leaf 3 (L3Base), 2 cm from the tip of leaf 3 (L3Tip), mature mid-leaf tissue sampled during mid-day (LMAD), mature mid-leaf tissue sampled during mid-night (LMAN), and developing kernels harvested after 350 growing degree days after pollination (Kern). The imputed expression from models was highly correlated between tissues when compared to the measured transcript expression. In all panels, closely related tissues like matured mid-leaf tissue expression sampled during mid-day (LMAD) and matured mid-leaf tissue expression sampled during mid-night (LMAD) were highly correlated.
(TIF)

S4 Fig Correlation distribution of *trans* components of expression between tissues. The different color lines in each panel represent 21 different combinations of the 7 different tissues as labeled on the right: germinating seedlings root (GRoot), germinating seedlings shoot (GShoot), 2 cm from the base of leaf 3 (L3Base), 2 cm from the tip of leaf 3 (L3Tip), mature mid-leaf tissue sampled during mid-day (LMAD), mature mid-leaf tissue sampled during mid-night (LMAN), and developing kernels harvested after 350 growing degree days after pollination (Kern). Similar to measured transcript expression, closely related tissues like matured leaf expression during the day (LMAD) and matured leaf expression during the night (LMAD) were highly correlated.
(TIF)

S5 Fig Haplotype associated RNA expression (HARE) was highly correlated across tissues as compared to measured transcript expression. Different colors represent HARE imputed from three statistical models: Model 1 (*cis* fixed effect), 2 (*cis* random effect), and 3 (*cis* + *trans* random effects), and measured transcript expression. The distribution is the pairwise correlation of ~8000 highly expressed genes across 21 different combinations from 7 different tissues.
(TIF)

S6 Fig Prediction accuracy using HARE from model 1, 2, and 3 (see Materials and methods) for predicting three different traits: Days to Anthesis (DTA), Days to Silking (DTS), and Plant Height (PH) using a) model trained in NAM and tested in Goodman b) model trained in Goodman and tested in NAM. The different symbols represent HARE from different tissues: germinating seedlings shoot (GShoot), developing kernels harvested after 350 growing degree days after pollination (Kern), 2 cm from the base of leaf 3 (L3Base), and mature mid-leaf tissue sampled during mid-day (LMAD).
(TIF)

S7 Fig Within-panel prediction accuracy in the Goodman panel using HARE (red dot), 100 random HARE (box plot), and measured expression (blue dot) from individual tissues or all tissues integrated as mean or maximum expression. Individual tissues included: germinating seedlings root (GRoot), germinating seedlings shoot (GShoot), 2 cm from the base of leaf 3 (L3Base), 2 cm from the tip of leaf 3 (L3Tip), mature mid-leaf tissue sampled during mid-day (LMAD), mature mid-leaf tissue sampled during mid-night (LMAN), and developing kernels harvested after 350 growing degree days after pollination (Kern). The model was trained in 80% of the panel and tested in the remaining 20%.
(TIF)

S8 Fig Change in prediction accuracy using HARE over the mean of random expression (blue dashed line) from five different tissues: germinating seedlings root (GRoot), 2 cm from the base of leaf 3 (L3Base), mature mid-leaf tissue sampled during mid-day (LMAD), mature mid-leaf tissue sampled during mid-night (LMAN), and developing kernels harvested after 350 growing degree days after pollination (Kern). Genomic prediction models were (a) trained in the Goodman panel and tested in 20 subsets of NAM, (b) trained in 20 subsets of NAM and tested in the Goodman panel. The subsets of NAM were generated by randomly selecting 10 genotypes from each family resulting in a total of 250 genotypes (see Materials and methods). Accuracy was averaged over the 20 random subsets before determining significance. The black shapes represent statistically significant differences at P-values <0.05 and red shapes represent no statistical significance. P-values were calculated using a Monte Carlo procedure.
(TIF)

S9 Fig Comparison of prediction accuracy within the Goodman Association panel using HARE, measured expression (measured_exp), and SNPs. The boxplot in HARE and measured expression are the accuracy from 7 diverse tissues: germinating seedlings root, germinating seedlings shoot, 2 cm from the base of leaf 3, 2 cm from the tip of leaf 3, mature mid-leaf tissue sampled during mid-day, mature mid-leaf tissue sampled during mid-night, and developing kernels harvested after 350 growing degree days after pollination. The model was trained in 80% of the panel and tested in the remaining 20%.
(TIF)

S10 Fig Prediction accuracy of 26 complex traits using SNPs. Different color symbols represent the accuracy from the model trained in Goodman Association panel and tested in NAM and trained in NAM and tested in Goodman Association panel.
(TIF)

S11 Fig Cross-panel prediction accuracy using SNPs and HARE from 7 tissues for models (a) trained in Goodman panel and tested in NAM, and (b) trained in NAM and tested in Goodman panel. (TIF)

S1 Table Prediction accuracy of 26 complex traits in the Goodman Association panel using HARE from 7 diverse tissues: germinating seedlings root (GRoot), germinating seedlings shoot (GShoot), 2 cm from the base of leaf 3 (L3Base), 2 cm from the tip of leaf 3 (L3Tip), mature mid-leaf tissue sampled during mid-day (LMAD), mature mid-leaf tissue sampled during mid-night (LMAN), and developing kernels harvested after 350 growing degree days after pollination (Kern), mean, and maximum expression of genes across all tissues. P value (high) and P value (low) were calculated using a Monte Carlo procedure to test if the accuracy using HARE was significantly higher or lower than random HARE. Models were trained in NAM and tested in Goodman Association panel.
(CSV)

S2 Table Prediction accuracy of 26 complex traits in NAM using HARE from 7 diverse tissues: germinating seedlings root (GRoot), germinating seedlings shoot (GShoot), 2 cm from the base of leaf 3 (L3Base), 2 cm from the tip of leaf 3 (L3Tip), mature mid-leaf tissue sampled during mid-day (LMAD), mature mid-leaf tissue sampled during mid-night (LMAN), and developing kernels harvested after 350 growing degree days after pollination (Kern), mean, and maximum expression of genes across all tissues. P value (high) and P value (low) were calculated using a Monte Carlo procedure to test if the accuracy using HARE was significantly higher or lower than random HARE. Models were trained in Goodman Association panel and tested in NAM.
(CSV)
