# Supplementary figures and images for: Haplotype associated RNA expression (HARE) improves prediction of complex traits in maize
Source: PLoS Genet. 2021 Oct 4;17(10):e1009568. doi: 10.1371/journal.pgen.1009568 (PMC8516254; doi:10.1371/journal.pgen.1009568)

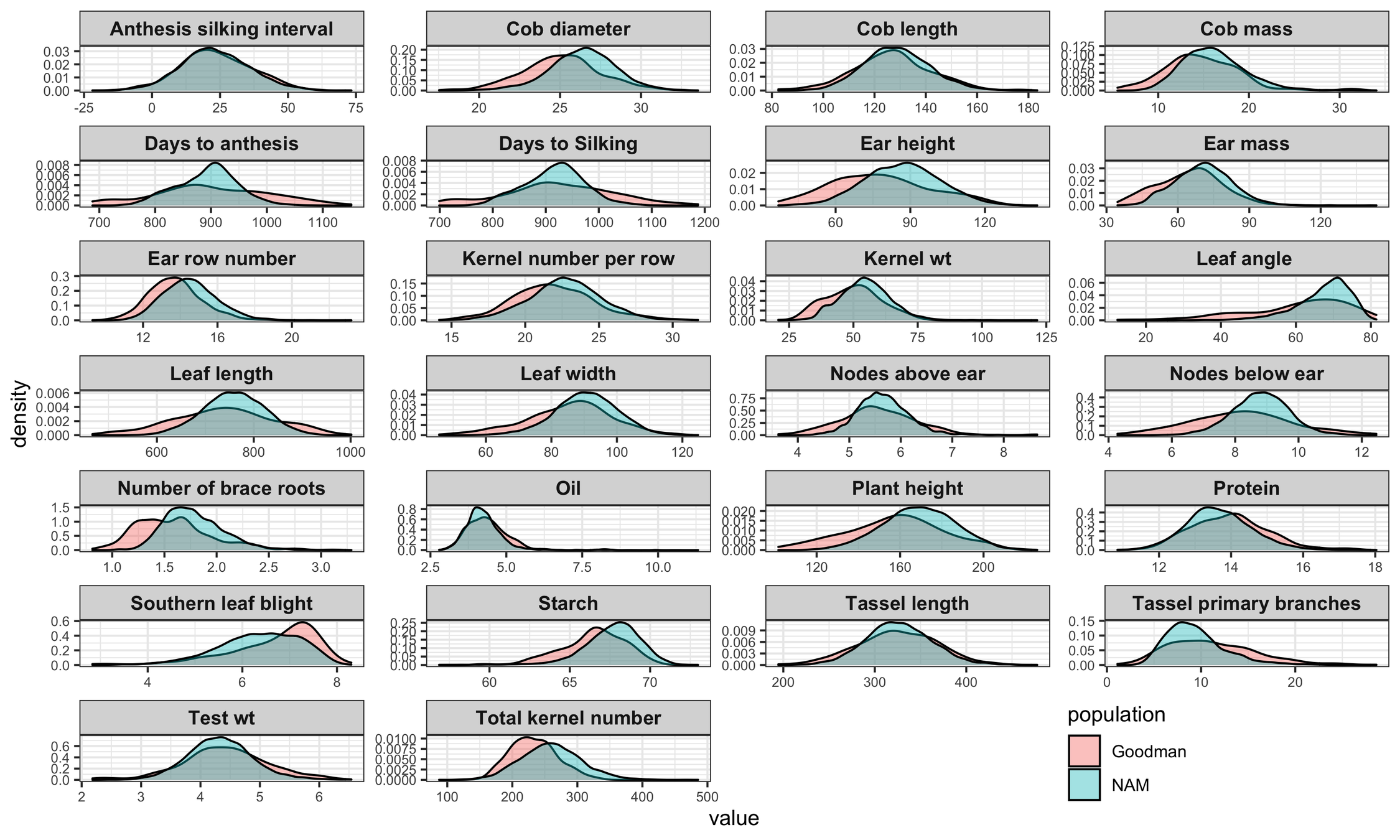

Supplement: S1 Fig — (TIF) [file pgen.1009568.s001.tif]

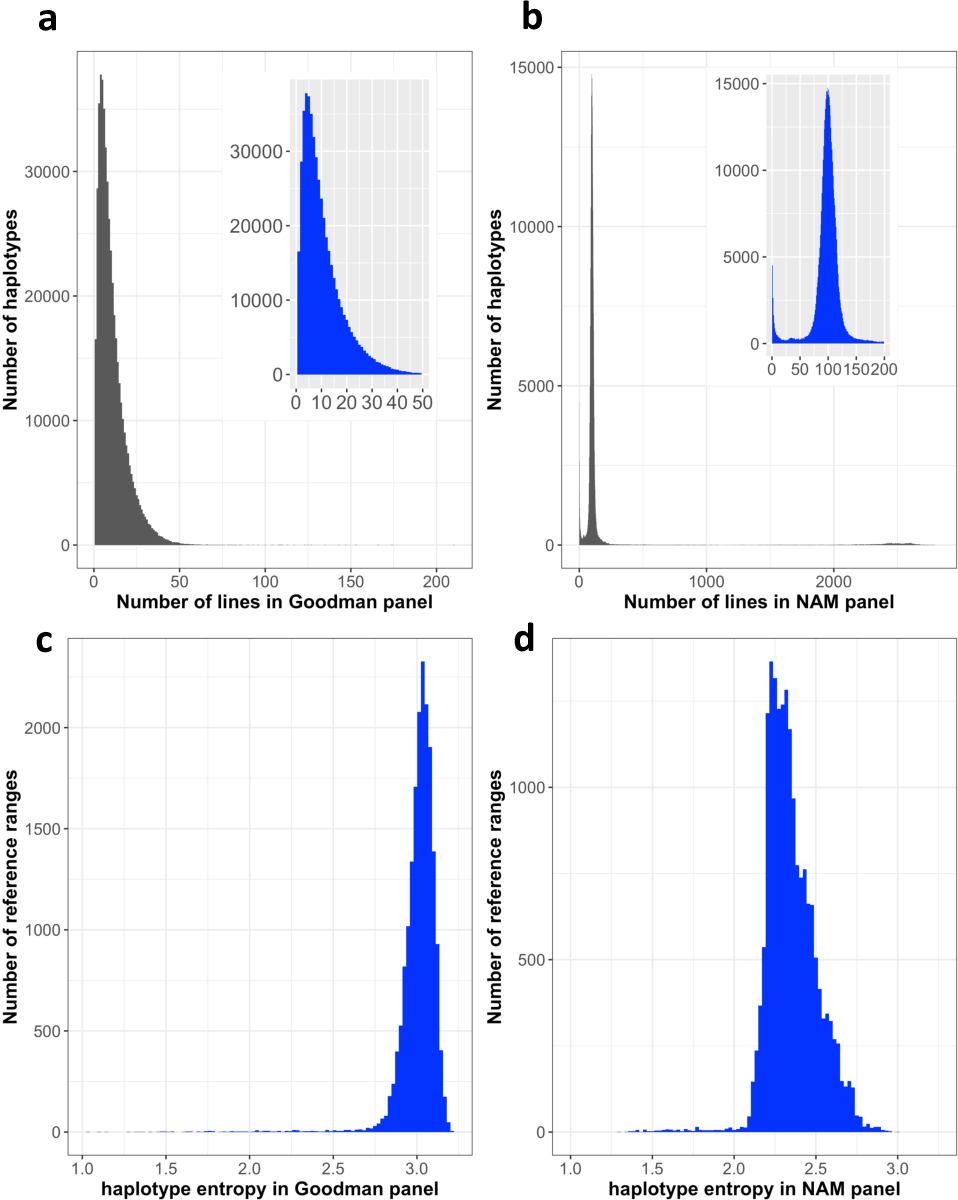

Supplement: S2 Fig — Median haplotype frequency were 8 and 100 in the Goodman and NAM, respectively, resulting in higher entropy in the Goodman panel as compared to NAM. Entropy was calculated from haplotype frequency in each reference region. (TIF) [file pgen.1009568.s002.tif]

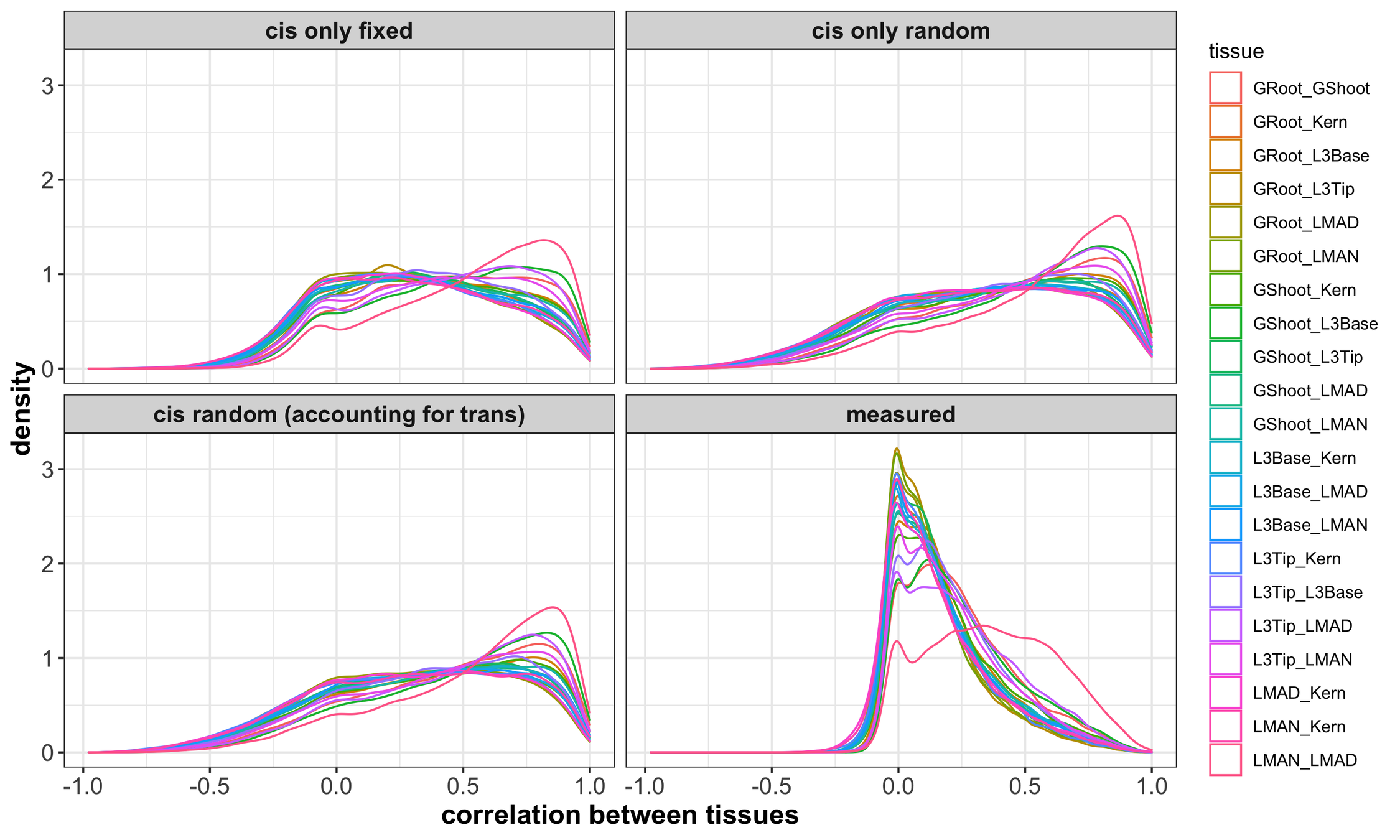

Supplement: S3 Fig — The different color lines in each panel represent 21 different combinations of the 7 different tissues as labeled on the right: germinating seedlings root (GRoot), germinating seedlings shoot (GShoot), 2 cm from the base of leaf 3 (L3Base), 2 cm from the tip of leaf 3 (L3Tip), mature mid-leaf tissue sampled during mid-day (LMAD), mature mid-leaf tissue sampled during mid-night (LMAN), and developing kernels harvested after 350 growing degree days after pollination (Kern). The imputed expression from models was highly correlated between tissues when compared to the measured transcript expression. In all panels, closely related tissues like matured mid-leaf tissue expression sampled during mid-day (LMAD) and matured mid-leaf tissue expression sampled during mid-night (LMAD) were highly correlated. (TIF) [file pgen.1009568.s003.tif]

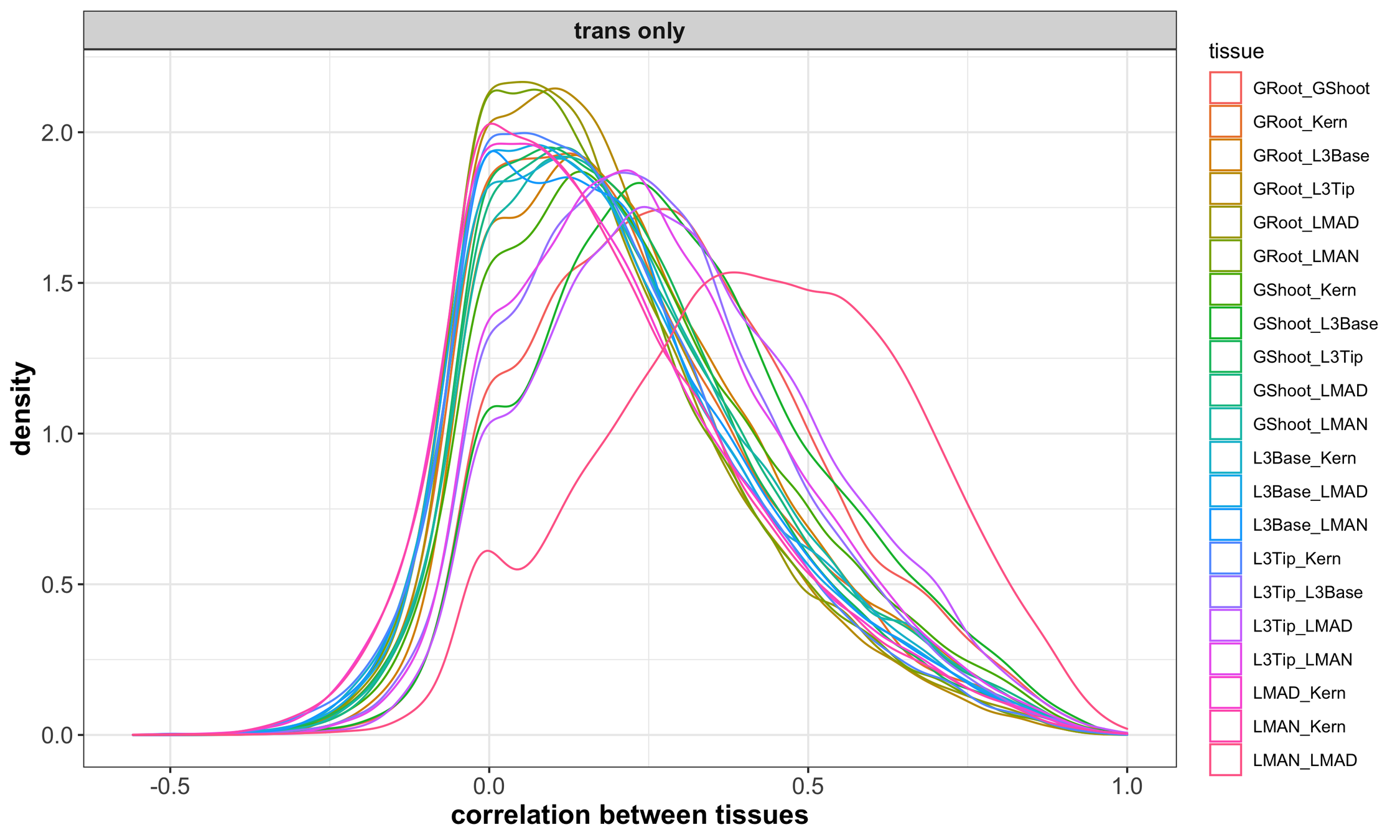

Supplement: S4 Fig — The different color lines in each panel represent 21 different combinations of the 7 different tissues as labeled on the right: germinating seedlings root (GRoot), germinating seedlings shoot (GShoot), 2 cm from the base of leaf 3 (L3Base), 2 cm from the tip of leaf 3 (L3Tip), mature mid-leaf tissue sampled during mid-day (LMAD), mature mid-leaf tissue sampled during mid-night (LMAN), and developing kernels harvested after 350 growing degree days after pollination (Kern). Similar to measured transcript expression, closely related tissues like matured leaf expression during the day (LMAD) and matured leaf expression during the night (LMAD) were highly correlated. (TIF) [file pgen.1009568.s004.tif]

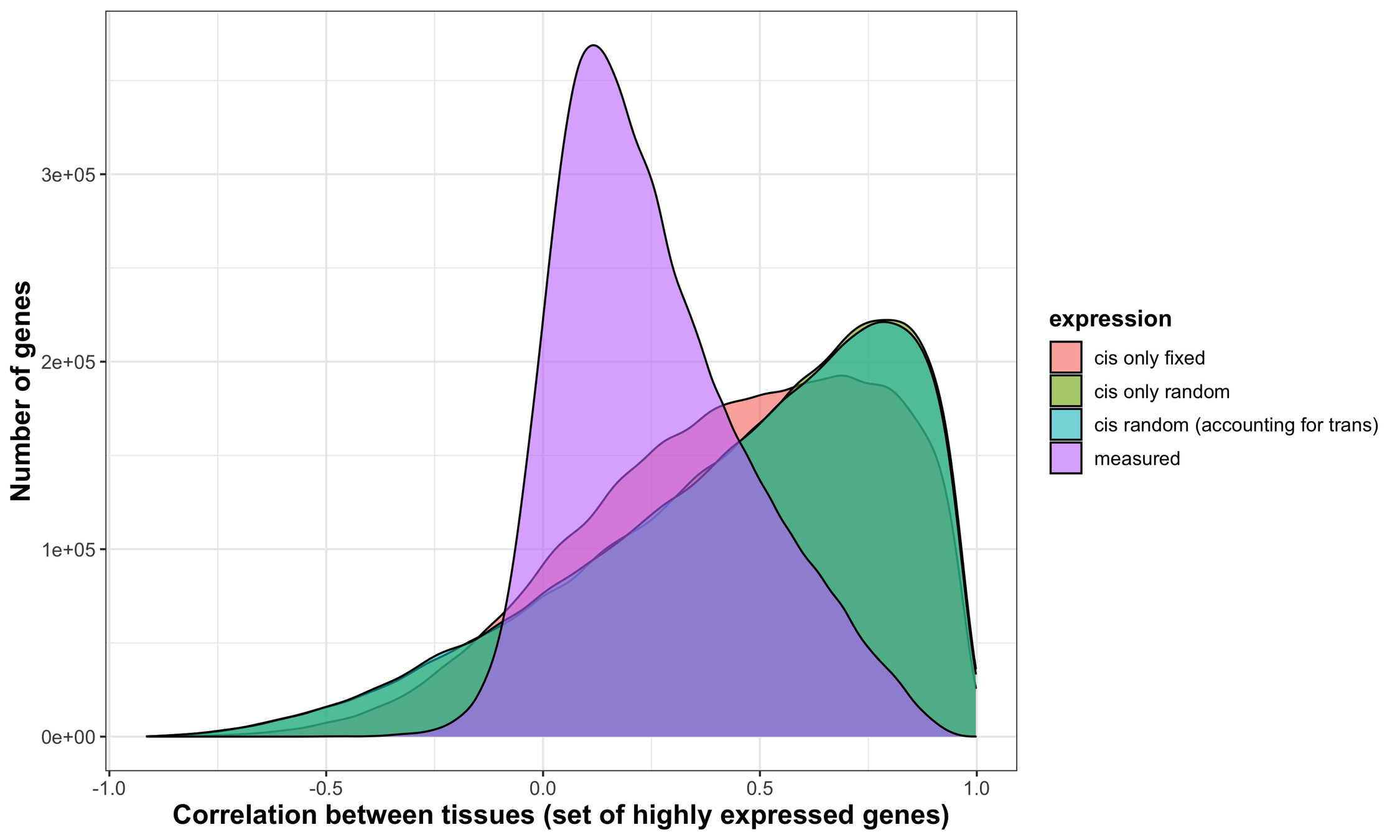

Supplement: S5 Fig — Different colors represent HARE imputed from three statistical models: Model 1 (cis fixed effect), 2 (cis random effect), and 3 (cis + trans random effects), and measured transcript expression. The distribution is the pairwise correlation of ~8000 highly expressed genes across 21 different combinations from 7 different tissues. (TIF) [file pgen.1009568.s005.tif]

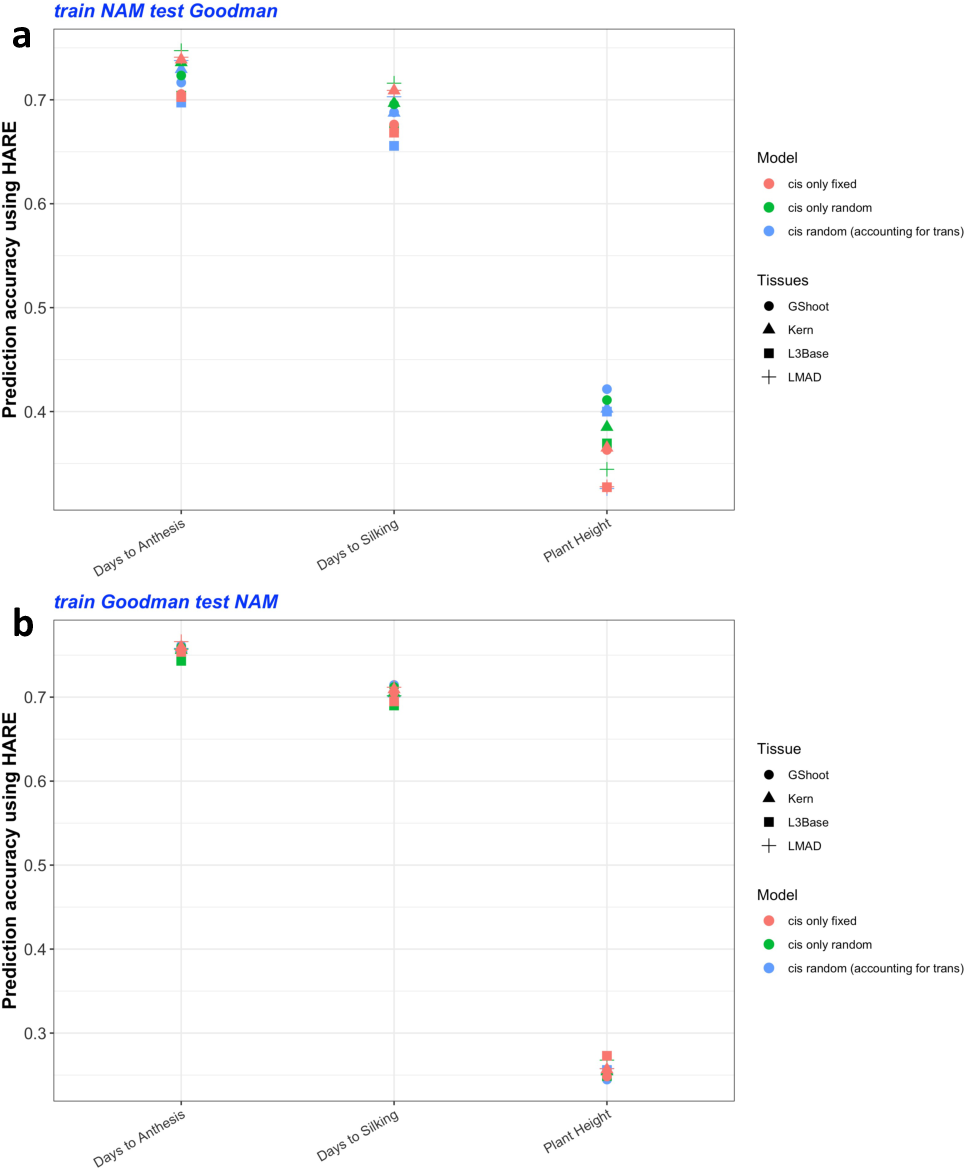

Supplement: S6 Fig — The different symbols represent HARE from different tissues: germinating seedlings shoot (GShoot), developing kernels harvested after 350 growing degree days after pollination (Kern), 2 cm from the base of leaf 3 (L3Base), and mature mid-leaf tissue sampled during mid-day (LMAD). (TIF) [file pgen.1009568.s006.tif]

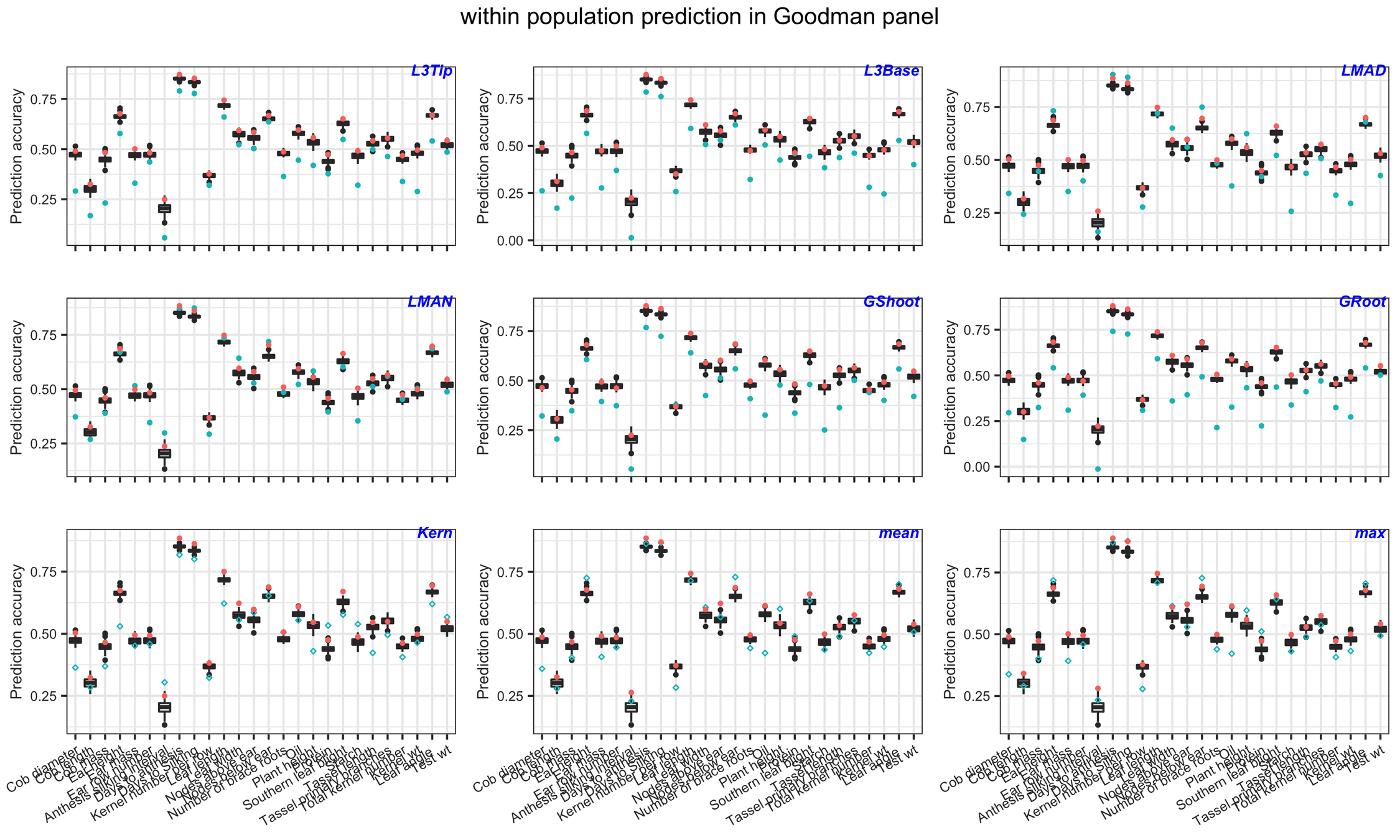

Supplement: S7 Fig — Individual tissues included: germinating seedlings root (GRoot), germinating seedlings shoot (GShoot), 2 cm from the base of leaf 3 (L3Base), 2 cm from the tip of leaf 3 (L3Tip), mature mid-leaf tissue sampled during mid-day (LMAD), mature mid-leaf tissue sampled during mid-night (LMAN), and developing kernels harvested after 350 growing degree days after pollination (Kern). The model was trained in 80% of the panel and tested in the remaining 20%. (TIF) [file pgen.1009568.s007.tif]

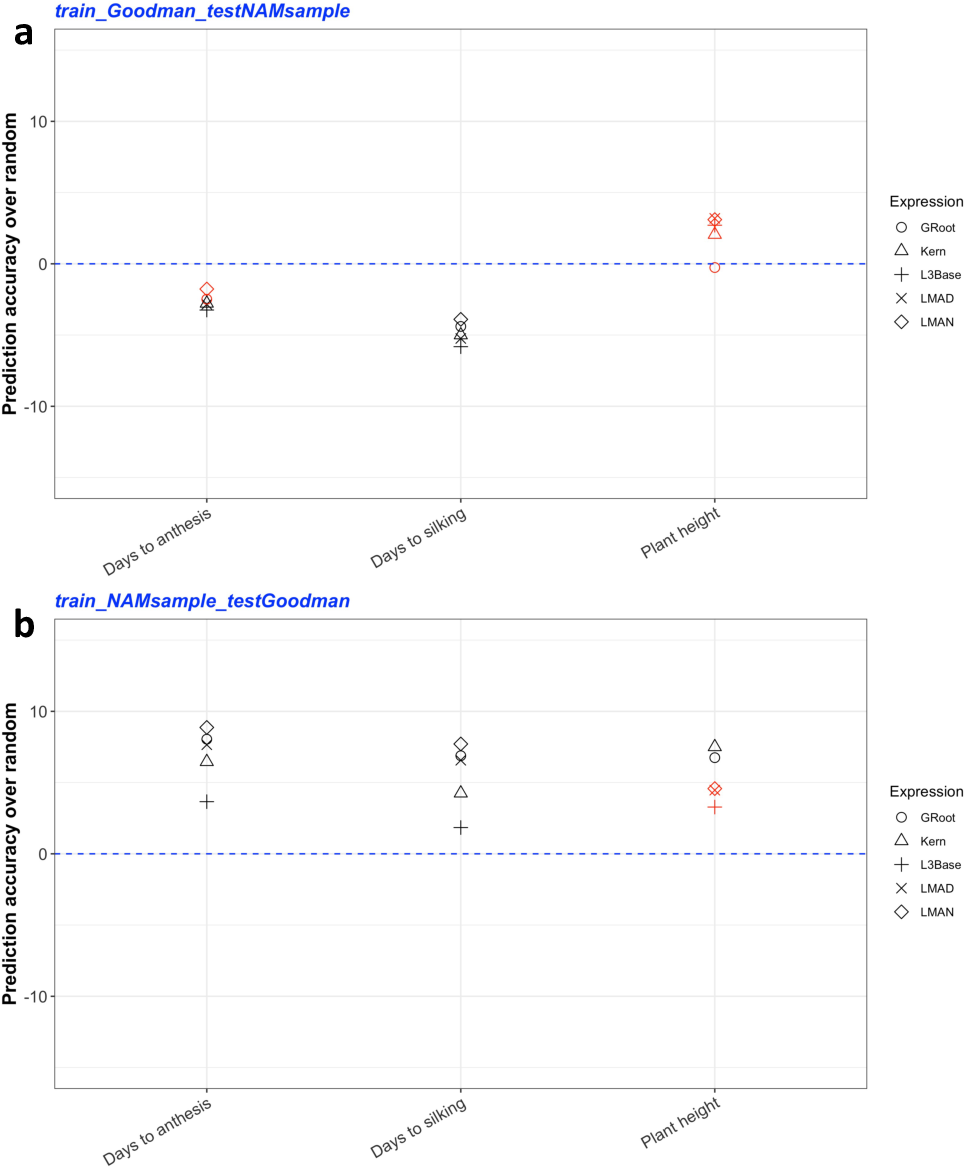

Supplement: S8 Fig — The subsets of NAM were generated by randomly selecting 10 genotypes from each family resulting in a total of 250 genotypes (see Materials and methods). Accuracy was averaged over the 20 random subsets before determining significance. The black shapes represent statistically significant differences at P-values <0.05 and red shapes represent no statistical significance. P-values were calculated using a Monte Carlo procedure. (TIF) [file pgen.1009568.s008.tif]

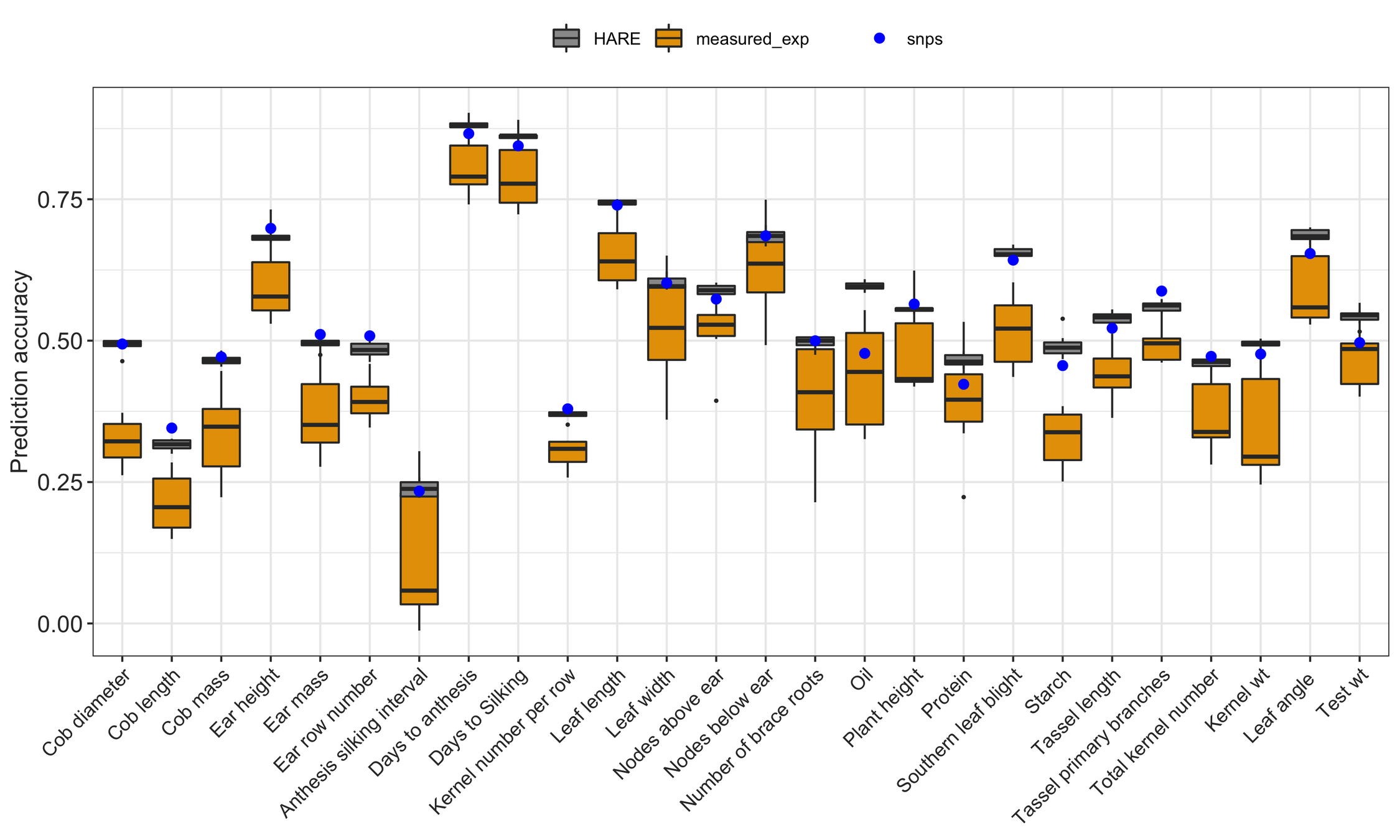

Supplement: S9 Fig — The boxplot in HARE and measured expression are the accuracy from 7 diverse tissues: germinating seedlings root, germinating seedlings shoot, 2 cm from the base of leaf 3, 2 cm from the tip of leaf 3, mature mid-leaf tissue sampled during mid-day, mature mid-leaf tissue sampled during mid-night, and developing kernels harvested after 350 growing degree days after pollination. The model was trained in 80% of the panel and tested in the remaining 20%. (TIF) [file pgen.1009568.s009.tif]

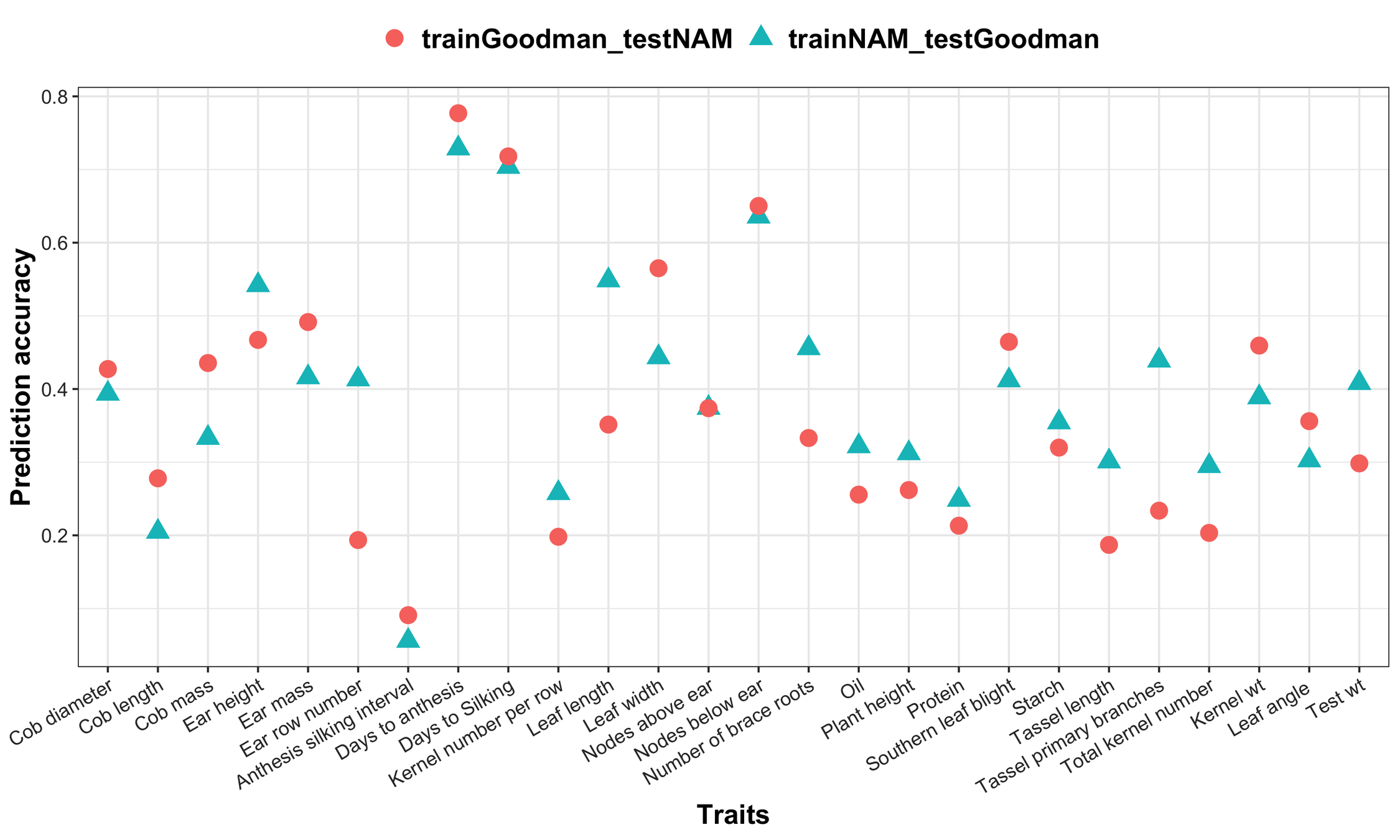

Supplement: S10 Fig — Different color symbols represent the accuracy from the model trained in Goodman Association panel and tested in NAM and trained in NAM and tested in Goodman Association panel. (TIF) [file pgen.1009568.s010.tif]

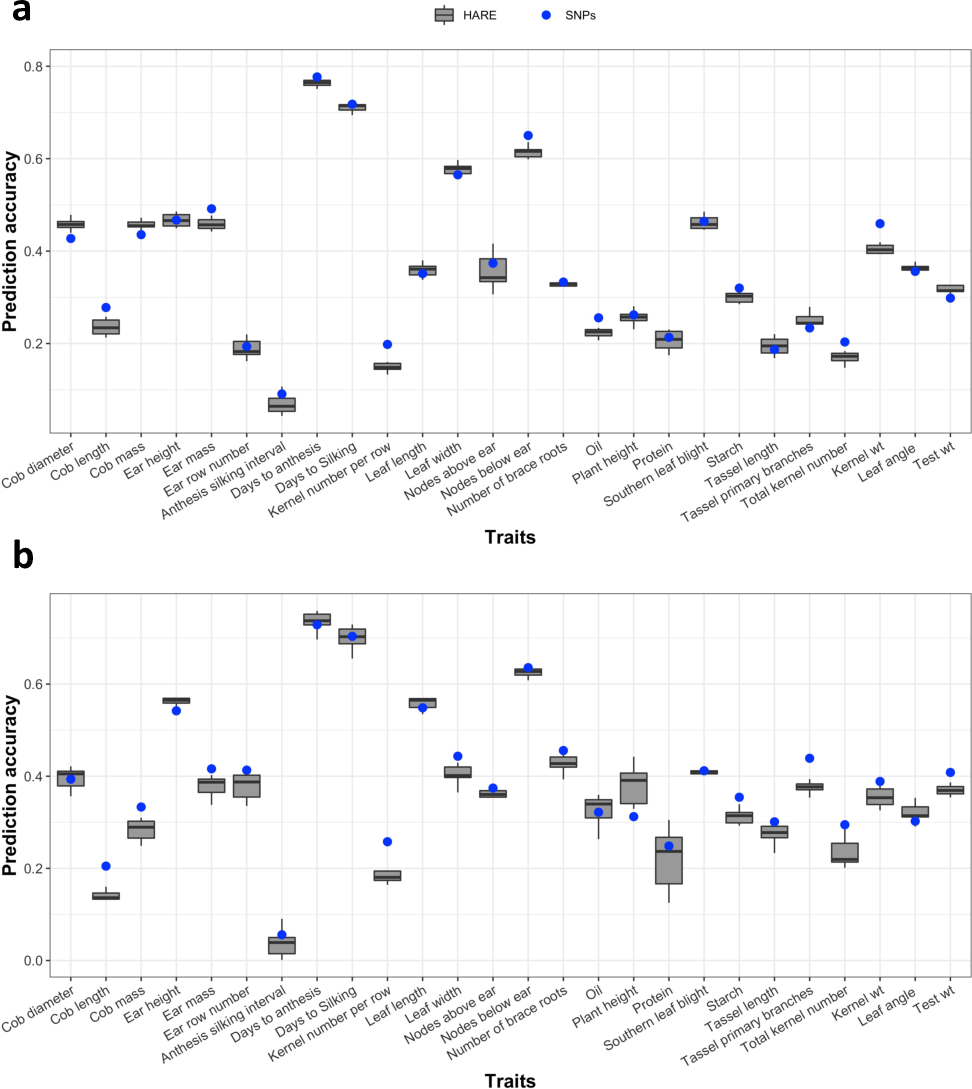

Supplement: S11 Fig — (TIF) [file pgen.1009568.s011.tif]
